# *In Vivo* Yeast Cell Morphogenesis Is Regulated by a p21-Activated Kinase in the Human Pathogen *Penicillium marneffei*

**DOI:** 10.1371/journal.ppat.1000678

**Published:** 2009-11-26

**Authors:** Kylie J. Boyce, Lena Schreider, Alex Andrianopoulos

**Affiliations:** Department of Genetics, University of Melbourne, Parkville, Victoria, Australia; Carnegie Mellon University, United States of America

## Abstract

Pathogens have developed diverse strategies to infect their hosts and evade the host defense systems. Many pathogens reside within host phagocytic cells, thus evading much of the host immune system. For dimorphic fungal pathogens which grow in a multicellular hyphal form, a central attribute which facilitates growth inside host cells without rapid killing is the capacity to switch from the hyphal growth form to a unicellular yeast form. Blocking this transition abolishes or severely reduces pathogenicity. Host body temperature (37°C) is the most common inducer of the hyphal to yeast transition *in vitro* for many dimorphic fungi, and it is often assumed that this is the inducer *in vivo*. This work describes the identification and analysis of a new pathway involved in sensing the environment inside a host cell by a dimorphic fungal pathogen, *Penicillium marneffei*. The *pakB* gene, encoding a p21-activated kinase, defines this pathway and operates independently of known effectors in *P. marneffei*. Expression of *pakB* is upregulated in *P. marneffei* yeast cells isolated from macrophages but absent from *in vitro* cultured yeast cells produced at 37°C. Deletion of *pakB* leads to a failure to produce yeast cells inside macrophages but no effect *in vitro* at 37°C. Loss of *pakB* also leads to the inappropriate production of yeast cells at 25°C *in vitro*, and the mechanism underlying this requires the activity of the central regulator of asexual development. The data shows that this new pathway is central to eliciting the appropriate morphogenetic response by the pathogen to the host environment independently of the common temperature signal, thus clearly separating the temperature- and intracellular-dependent signaling systems.

## Author Summary

Dimorphic fungal pathogens pose significant health and agricultural problems worldwide. These fungi have the capacity to switch between a multicellular hyphal growth form and a unicellular yeast growth form. Often one form is pathogenic, found in infected hosts, and the other is not. Many dimorphic fungal pathogens of humans produce the yeast form during infection and this form resides within host phagocytic immune cells, where it can tolerate killing by these cells and is not exposed to the acquired immune system. Inhibiting the pathogen's ability to switch growth forms has been shown to block pathogenicity. This study identifies a pathway used by the fungal pathogen to sense the host and switch to the appropriate growth form. This study provides new insights into the molecular mechanisms which are important for pathogenicity and may identify factors which can be targeted to block the ability of the pathogen to successfully reside within host cells.

## Introduction

Host immune systems actively survey and attempt to kill invading pathogens, so for pathogens to successfully infect a host the pathogen must be able to evade or tolerate these systems. A number of pathogens enter phagocytic cells and primarily reside within these cells to avoid the host's immune defense system. To continually reside within phagocytic cells of the immune system without disrupting their integrity, pathogens such as fungi which can grow in a filamentous, multicellular hyphal form, must be able to produce a uninucleate yeast growth form. The ability to switch between the filamentous and yeast forms is a tightly regulated process known as dimorphic switching. Dimorphism has been shown to be a critical pathogenicity determinant.

*Penicillium marneffei* exhibits dimorphic switching and hence can grow in two distinct cellular forms; multicellular hyphae and unicellular yeast. *P. marneffei* is the only known *Penicillium* species which is dimorphic and the switch between growth forms is regulated by temperature [1]. At 25°C, in the saprophytic growth phase, *P. marneffei* grows as multinucleate, septate, branched hyphae. These hyphae produce conidia, the infectious agent, from specialized multicellular structures termed conidiophores. When switched to 37°C, *P. marneffei* undergoes a developmental process termed arthroconidiation. Cellular and nuclear division become coupled, double septa are laid down and hyphae fragment at these septation sites to liberate uninucleate yeast cells which subsequently divide by fission [1]. The yeast cells are the pathogenic form and multiple yeast cells are seen in the pulmonary alveolar macrophages and peripheral blood mononuclear cells of infected individuals [2]. *P. marneffei* infection is likely to occur through inhalation of the conidia produced by the filamentous saprophytic form [2]. It has been proposed that the conidia bind to laminin in the bronchoalveolar epithelia via a sialic acid-specific lectin [3],[4]. The conidia are then ingested by host pulmonary alveolar macrophages where they germinate into unicellular yeast cells which divide by fission. Therefore the ability to produce infectious propagules such as asexual spores (conidiation) in the saprophytic growth state and the capacity upon infection to switch between a multicellular hyphal growth form and a unicellular yeast pathogenic form are both crucial for pathogenicity.

Polarity establishment is necessary for the differentiation of distinct cell types during development. The Rho GTPases Cdc42 and Rac act as molecular switches to localize or activate proteins associated with polarized growth. The *CDC42* homologue in *P. marneffei, cflA*, is required for germination of conidia at both 25°C and 37°C, polarized growth and division of hyphae at 25°C and for polarized growth of yeast cells at 37°C [5]. The *P. marneffei* genome also encodes a second Rac-like Rho GTPase, *cflB*. Similar to *cflA*, *cflB* is required for the polarized growth and division of hyphae at 25°C [6]. However, unlike *cflA*, *cflB* plays a key role during asexual development (conidiation) at 25°C and is not required for the polarized growth of yeast cells at 37°C [6]. In *Saccharomyces cerevisiae*, the Rho GTPase Cdc42p activates the p21 activated kinases (PAKs) Ste20p and Cla4p [7]–[11]. *P. marneffei* possesses both *STE20* and *CLA4* homologues; *pakA* (*STE20*) and *pakB* (*CLA4*). Characterization of *pakA* in *P. marneffei* has shown that this gene is essential for conidial germination at 37°C and polarized growth of yeast cells, acting downstream of both a heterotrimeric G protein and Cdc42 pathway [12]. Δ*pakA* and *pakA* strains containing a mutation in the conserved Cdc42/Rac Interactive Binding (CRIB) domain (*pakA^H108G^*) fail to germinate into pathogenic yeast cells *in vivo* [12].

This study describes the characterization of the second PAK in *P. marneffei*, PakB. The *pakB* gene is expressed during hyphal growth and asexual development at 25°C and is essential for the generation of these 25°C-specific cell types. Deletion of *pakB* results in yeast-like morphology and the inappropriate production of yeast cells at 25°C. Deletion of the primary regulator of asexual development, *brlA*, in the Δ*pakB* strain results in suppression of this inappropriate yeast cell production suggesting that these yeast cells are dependent on the conidiation program. PakB is also essential for yeast morphogenesis during infection but not *in vitro*. Macrophages infected with the Δ*pakB* strain exhibit highly branched, septate, hyphal cell growth but no yeast cells. These results suggest that the developmental pathways regulating conidiation at 25°C and yeast cell production at 37°C share a number of regulatory components including PakB and that the developmental outcome of each pathway is regulated in part by the mode of cellular division.

## Results

### Cloning the *CLA4* p21-activated kinase orthologue from *P. marneffei*

A previous low stringency hybridisation screen of a *P. marneffei* genomic library using an *Aspergillus nidulans* sequence with strong homology to *S. cerevisiae* Ste20p yielded five positive clones, which fell into two classes based on restriction enzyme digestion patterns [12]. Sequencing of a cloned fragment from one of these classes (pKB5751) revealed strong sequence homology to *STE20*-like PAKs and the gene within this clone was subsequently named *pakA* [12]. A fragment from the second class of clones was also subcloned (pKB4904) and sequencing revealed strong sequence homology to *CLA4*-like PAKs from *Candida albicans* (48% identity, 58% similarity), *Ashbya gossypii* (49% identity, 57% similarity), *Ustilago maydis* (53% identity, 61% similarity) and *S. cerevisiae* (48% identity, 57% similarity) (Figure S1). The gene within this clone was named *pakB*. The homology is to a large extent restricted to the CRIB and kinase domains where, for example, PakB shows 74% and 76% identity, respectively, to the same domains in *U. maydis* Cla4. The predicted PakB protein exhibits 37% identity and 46% similarity to PakA.

The predicted PakB protein is 733 amino acids in length and contains a PH domain at 81–191, a Cdc42/Rac Interactive Binding (CRIB) domain (also called PBD for p21-Rho-binding domain) at positions 195-256 and a predicted kinase domain at 443–712 (http://pfam.sanger.ac.uk/). An 11 amino acid sequence in the non-catalytic C-terminal region of *S. cerevisiae* Ste20p has been shown to be required for interaction with Ste4p, the beta subunit of a heterotrimeric G protein, during pheromone signaling [13],[14]. This region in *S. cerevisiae* Cla4p has also been shown to interact weakly with Ste4p [13]. The consensus sequence SSLφPLI/VXφφβ (where X is any residue, φ for A, I, L, S or T and β is for basic residues) is also found in *P. marneffei* PakA (619–629).

**Figure 1:**
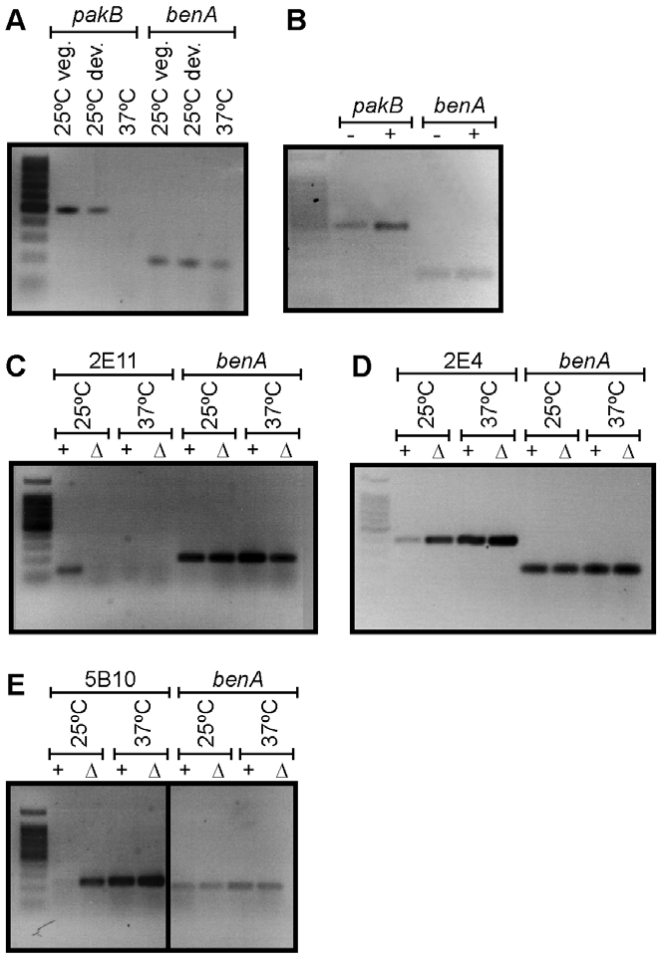
*pakB* is expressed predominately at 25°C and deletion results in inappropriate expression of hyphal specific and yeast specific genes at 25°C. (A) Expression of *pakB* shown by RT PCR on RNA isolated from vegetative hyphae grown for 2 days in liquid medium at 25°C (25°C veg.), asexual development cultures grown for 4 days on solid medium at 25°C (25°C dev.) and yeast cells grown for 6 days in liquid medium at 37°C (37°C). A *benA* loading control is shown. *pakB* is expressed at the highest level in vegetative hyphae at 25°C and to a lesser extent during asexual development at 25°C. Expression of *pakB* is barely detectable at 37°C. (B) RT PCR performed on RNA isolated from wildtype *P. marneffei* grown in the absence (−) or presence (+) of LPS activated murine macrophages after 24 hrs. In the infection process, macrophages were incubated for 2 hours with wildtype conidia to allow for phagocytosis before washing to remove non-phagocytosed conidia. Therefore, all *P. marneffei* cells are intracellular after 24 hrs. The *benA* loading control is shown. Expression of *pakB* is increased during macrophage infection. (C–E) RT PCR on RNA isolated from both the wildtype (+) and Δ*pakB* (Δ) strains grown as vegetative hyphae grown for 2 days in liquid medium at 25°C (25°C) and as yeast cells grown for 6 days in liquid medium at 37°C (37°C). The *benA* loading controls are shown. (C) In wildtype, the hyphal specific probe 2E11 is expressed specifically in hyphae at 25°C and not in yeast cells at 37°C. The 2E11 transcript is not detectable in the Δ*pakB* strain at 25°C. (D) In wildtype, 2E4 is expressed at a low level at 25°C and expression is greatly increased at 37°C. Expression of 2E4 is increased in the Δ*pakB* strain at 25°C. The amount of 2E4 expression is also slightly increased in the Δ*pakB* strain at 37°C. (E) The transcript of the yeast specific probe 5B10 is not detected in wildtype at 25°C, whereas, expression is high at 37°C. 5B10 is highly expressed in the Δ*pakB* strain at 25°C. The amount of 5B10 expression is also slightly increased in the Δ*pakB* strain at 37°C.

RNA was isolated from vegetative hyphae grown for 2 days in liquid medium at 25°C, asexual development (conidiation) cultures grown for 4 days on solid medium at 25°C and yeast cells grown for 6 days in liquid medium at 37°C. The level of *pakB* expression varied substantially depending on cell type. The level of *pakB* transcript was highest during vegetative hyphal growth at 25°C, lower during asexual development at 25°C and barely detectable during vegetative yeast growth at 37°C relative to the *benA* control (Figure 1A).

*P. marneffei* infection is believed to occur by inhalation of conidia, which bind to the laminin in the bronchoalveolar epithelium [2]–[4]. Conidia are then ingested by pulmonary alveolar macrophages and germinate directly into uninucleate yeast cells which proliferate within the macrophage [1]. To examine if *pakB* is expressed during infection, RNA was isolated from yeast cells derived either from LPS activated J774 murine macrophages at 37°C infected with wildtype conidia 24 hours post-infection or from yeast cells incubated in macrophage growth media at 37°C for 24 hours (Materials and Methods). Substantial levels of *pakB* expression were detected in cells isolated from infected macrophages suggesting that *pakB* expression is induced during infection (Figure 1B). Low levels of *pakB* expression was detected in yeast cells derived from the macrophage medium control (Figure 1B).

### PakB is localized to the hyphal apex, conidiophores and septation sites

**Figure 2:**
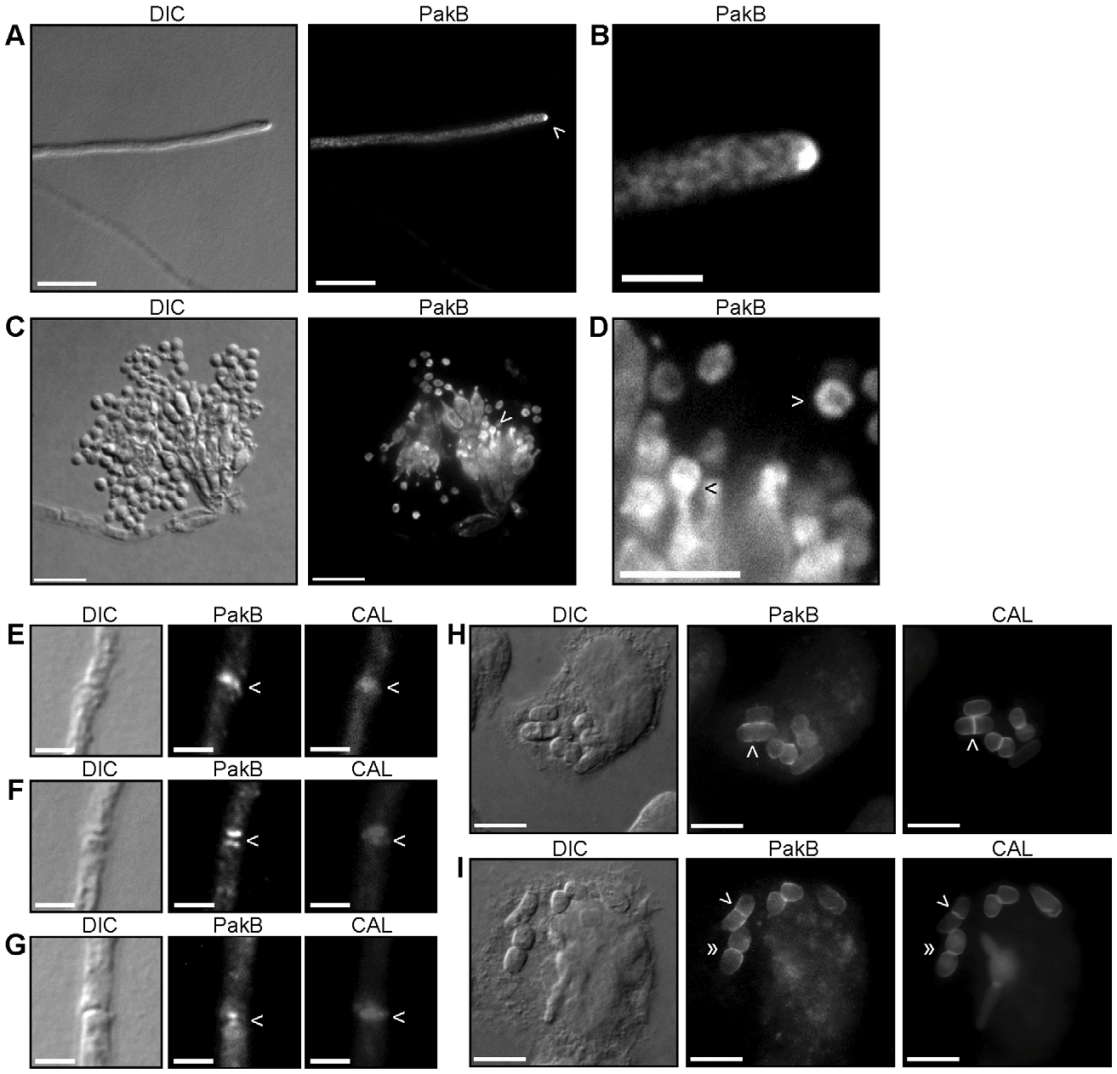
PakB localization during vegetative growth and asexual development at 25°C and during macrophage infection at 37°C. *pakB^+^* HA strains were grown on ANM + (NH_4_)_2_SO_4_ for 4 days at 25°C (A–G) or in LPS activated J774 murine macrophages for 24 hours (H–I) and immunofluorescently labeled with 3F10 rat monoclonal anti-HA primary and an ALEXA 488 goat anti-rat secondary antibody (PakB) and stained with calcofluor (CAL). (A) PakB is concentrated at the hyphal apex, indicated by the white arrowhead. (B) Magnification of region indicated by arrowhead in (A). (C) PakB is localized throughout all of the cell types of the conidiophore; metulae, phialides and newly formed conidia. (D) Magnification of region indicated by the white arrowhead in (C). PakB is concentrated at the phialide to conidium interface (black arrowhead) and around the periphery of newly formed conidia (white arrowhead). PakB is not visible in older conidia. (E–G) PakB is localized to nascent septation sites where it is co-localized with calcofluor stained septa, as a single band (E), as two bands on either side of the calcofluor stained septa (F) or as two spots on either side of the calcofluor stained septa (G). (H–I) During macrophage infection, PakB is localized around the yeast cell periphery. (H) PakB is not observed localized either at nascent septation sites prior to, or immediately after (white arrowheads), cell wall deposition. (I) Localization at septation sites can be observed prior to cell separation *in vivo* (white arrowheads). PakB is localized to the division site and to the region immediately adjacent during cell separation *in vivo* (double white arrowheads). Scale bars, 20 µm (A,C, H–I), 10 µm (D) and 2.5 µm (B, E–G).

To investigate the localization of PakB, a triple HA tag was inserted into a non-conserved region of PakB between the CRIB and kinase domains. The *pakB^+^* HA construct was co-transformed with the *barA^+^* gene into the *P. marneffei* strain G487 (*niaD pyrG areA^−^*). Transformants were selected for glufosinate resistance and confirmed by Southern blot analysis of genomic DNA (Materials and Methods). Anti-HA immunostaining was performed on two of the *pakB^+^* HA strains after 4 days growth at 25°C. PakB was observed concentrated at the hyphal apex (Figure 2A–B) and also localized to all of the cell types of the conidiophore (Figure 2C). PakB was particularly concentrated at the phialide to conidium interface and around the periphery of newly formed, but not old, conidia (Figure 2D). In addition, PakB was co-localized at nascent septation sites presenting either as a single band colocalised with calcofluor stained septa (Figure 2E), two bands on either side of the calcofluor stained septa (Figure 2F) or two spots on either side of the calcofluor stained septa (Figure 2G). PakB was not observed at older septa (data not shown).

To investigate the localization of *pakB* during infection, LPS activated J774 murine macrophages were infected with the *pakB^+^* HA strains and anti-HA immunostaining and calcofluor staining were performed 24 hours post-infection (Materials and Methods). PakB was localized around the cell periphery (Figure 2H–I). PakB was not localized either at nascent septation sites prior to, or immediately after, cell wall deposition (indicated by calcofluor staining) (Figure 2H). Weak localization at septation sites could be observed prior to cell separation (Figure 2I). PakB was localized at, and adjacent to, the division site during cell separation (Figure 2I).

### Deletion of *pakB* results in inappropriate yeast-like growth at 25°C

A construct in which a region encompassing the *pakB* coding sequence (+323 to +2618) was replaced with the *pyrG* selectable marker was used to create a *pakB* deletion strain. *P. marneffei* strain G487 (*niaD pyrG areA^−^*) was transformed with this construct and *pyrG^+^* transformants selected. Genomic DNA from the PyrG^+^ transformants was screened by Southern blotting to identify strains which possessed a restriction pattern consistent with replacement of *pakB* by *pyrG* at the genomic locus (data not shown). To generate a Δ*pakB pyrG^−^* strain, a Δ*pakB::pyrG^+^* deletion strain was plated on medium containing 5-fluoroorotic acid (5-FOA) (Materials and Methods). This strain was cotransformed with plasmids containing *pakB^+^* and *pyrG^+^* genes and co-transformants confirmed by Southern blot analysis. The Δ*pakB pakB^+^* transformants contained 2–8 copies of *pakB*.

**Figure 3:**
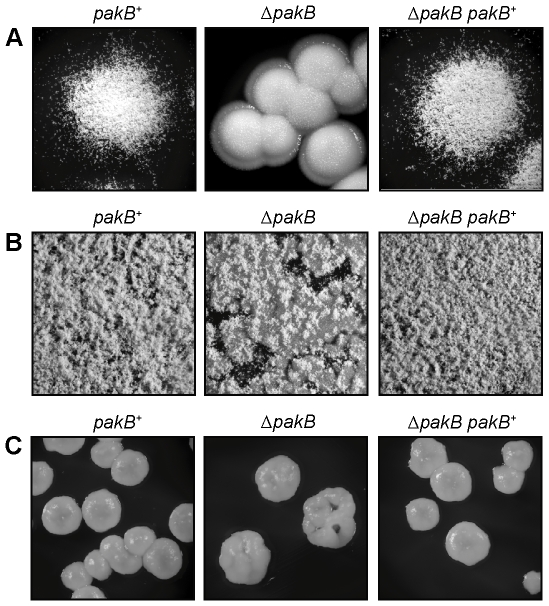
Deletion of *pakB* results in inappropriate yeast-like growth at 25°C. The *pakB^+^*, Δ*pakB* and Δ*pakB pakB^+^* strains were grown on ANM + (NH_4_)_2_SO_4_ for 10 days (A) or 14 days (B) at 25°C or on SD + (NH_4_)_2_SO_4_ for 5 days at 37°C (C). (A) Colonies of the *pakB* strains at 25°C (16× magnification). Wildtype colonies appear fuzzy due to the presence of radial, polarized hyphae growing along the agar surface and aerial hyphae. The hyphae are covered with asexual structures (conidiophores). The Δ*pakB* strain produces compact, mucoid, yeast-like colonies at 25°C. Colonies of the Δ*pakB* strain are not covered with conidiophores after 10 days growth. The Δ*pakB pakB^+^* strain is indistinguishable from wildtype. (B) After 14 days, the colony surface of wildtype (*pakB^+^*) shows many conidiophore structures (20× magnification). In contrast to wildtype, the Δ*pakB* strain produces fewer conidiophores and these are unevenly dispersed over the yeast-like colonial surface. The Δ*pakB pakB^+^* strain is indistinguishable from wildtype. (C) Colonies of the *pakB* strains at 37°C (32× magnification). Wildtype yeast colonies are compact and mucoid. The Δ*pakB* and Δ*pakB pakB^+^* strains are indistinguishable from wildtype at 37°C.

After 10 days at 25°C, wildtype *P. marneffei* grows as polarized vegetative hyphae which bear asexual structures (conidiophores). Colonies appear fuzzy and the surface is green due to the presence of pigmented asexual spores (conidia) on conidiophores (Figure 3A–B). The Δ*pakB* strain produced compact, mucoid yeast-like colonies after 10 days growth at 25°C which resemble the yeast colonies produced by the wildtype at 37°C (Figure 3A versus C). Despite this the Δ*pakB* strain conidiated upon longer incubation at 25°C (14 days). Conidiophore structures were visible under higher magnification, however, these were unevenly dispersed over the yeast-like colonies and not as profuse as in wildtype (Figure 3B). Transformation of the Δ*pakB* strain with *pakB^+^* (Δ*pakB pakB^+^*) completely restored the wildtype phenotype (Figure 3).

### Deletion of *pakB* results in inappropriate expression of hyphal specific and yeast specific genes at 25°C

Deletion of *pakB* resulted in yeast-like growth at 25°C (Figure 3A). To investigate the molecular basis underpinning this phenotype RT PCR analysis was performed to see if it correlates with a decrease in the expression of hyphal specific genes or an increase in the expression of yeast specific genes. RNA was isolated from both the wildtype and the Δ*pakB* strain grown as vegetative hyphae for 2 days in liquid medium at 25°C and as yeast cells for 6 days in liquid medium at 37°C and used for RT PCR with primers for a number of cell type specific genes (Canovas and Andrianopoulos, unpublished). In wildtype, the 2E11 probe was expressed specifically in hyphae at 25°C and not in yeast cells at 37°C (Figure 1C). In contrast, the 2E11 transcript was not detectable in the Δ*pakB* strain at 25°C (Figure 1C). In wildtype, the 2E4 transcript was expressed at a low level at 25°C and expression was greatly increased at 37°C (Figure 1D) while expression of 5B10 in wildtype was not detectable at 25°C but was high at 37°C (Figure 1E). Both 2E4 and 5B10 transcripts were highly expressed in the Δ*pakB* strain at 25°C (Figure 1D and E). The amount of 2E4 and 5B10 transcript was also slightly increased in the Δ*pakB* strain at 37°C (Figure 1D and E). This suggests that in the Δ*pakB* strain both a decrease in the expression of hyphal specific genes and an increase in the expression of yeast specific genes may contribute to the yeast-like growth phenotype at 25°C.

### The Δ*pakB* strain inappropriately produces yeast cells at 25°C

**Figure 4:**
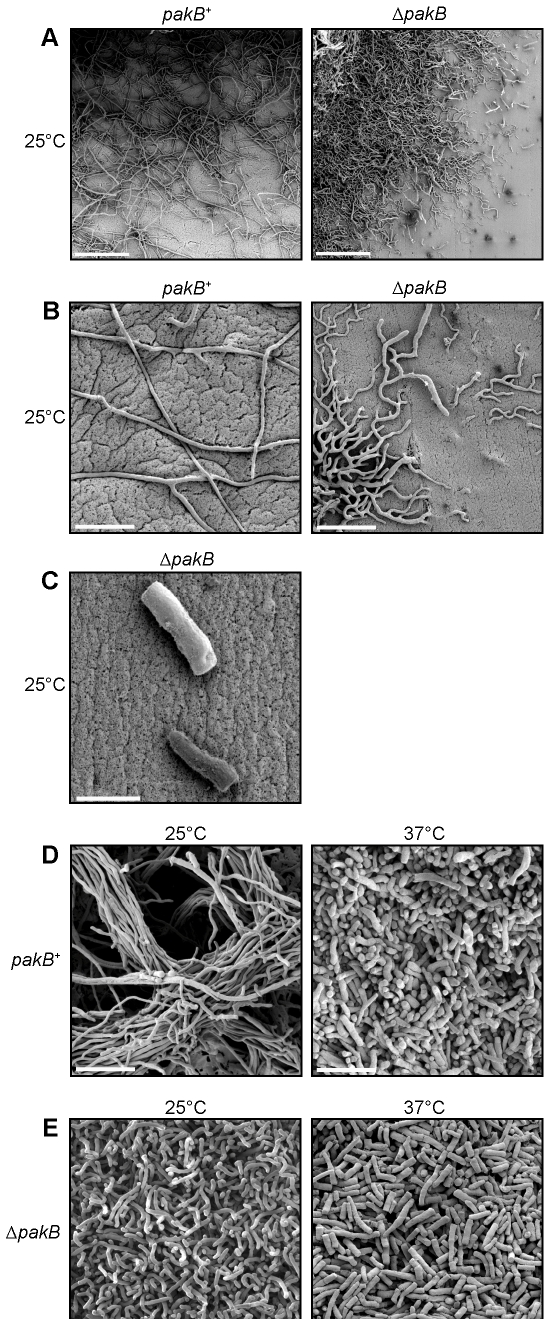
The Δ*pakB* strain displays yeast-like invasive growth and inappropriately produces yeast cells at 25°C. SEM of wildtype (*pakB* ^+^) and Δ*pakB* strains grown on ANM + (NH_4_)_2_SO_4_ for 10 days at 25°C (A–C), 10 days (25°C) or 5 days (37°C) on BHI. (A) Wildtype colonies are comprised of entangled, branched hyphae which radiate from the central mycelium. The edges of the colony are not clearly defined. In contrast, the colonies of the Δ*pakB* strain are compact and the colony edges are distinct. (B) Wildtype (*pakB* ^+^) hyphae grow along the surface of the agar in a polarized fashion. The hyphae of the Δ*pakB* strain are highly branched compared to wildtype and often invade the agar surface. (C) Individual yeast cells are observed around the Δ*pakB* colony periphery at 25°C. (D) After 10 days at 25°C on BHI, wildtype (*pakB* ^+^) colonies are comprised of large bundles of branched hyphae. After 5 days at 37°C, the wildtype colony is comprised of a mass of fragmented hyphae and yeast cells. (E) After 10 days at 25°C on BHI, the colony surface of the Δ*pakB* strain resembles that of wildtype at 37°C (D). The Δ*pakB* strain appears to produce more yeast cells than wildtype at 37°C. Scale bars, 100 µm (A), 20 µm (B, D and E) and 5 µm (C).

In order to characterize the cellular basis behind the yeast-like colonial morphology of the Δ*pakB* strain at 25°C, scanning electron microscopy (SEM) was performed on both wildtype and Δ*pakB* strains after 10 days growth at 25°C (Materials and Methods). Wildtype colonies appeared as a mass of entangled, branched hyphae which radiated from the centre of the colony in a polarized fashion (Figure 4A). The edges of the colony were not clearly defined due to countless hyphae which extended great distances from the colony periphery (Figure 4A). In contrast, the colonies of the Δ*pakB* strain were compact and therefore the colony edges were distinct (Figure 4A). The hyphae of the Δ*pakB* strain were more branched than wildtype and often invaded the agar surface (Figure 4B). Invasive growth is a common characteristic of wildtype arthroconidial growth at 37°C (not shown). In contrast to wildtype, in which yeast cells are never seen at 25°C, individual yeast cells were also observed around the Δ*pakB* colony periphery (Figure 4C). The wildtype and Δ*pakB* strains were also grown on the standard 37°C media, brain heart infusion (BHI), for 10 days at 25°C and for 5 days at 37°C. SEM of the colony surface showed wildtype colonies at 25°C were comprised of large bundles of branched hyphae (Figure 4D). At 37°C, wildtype undergoes arthroconidiation, a developmental process where hyphae fragment to liberate yeast cells which subsequently divide by fission. After 5 days at 37°C, the wildtype colony was comprised of a mass of fragmented hyphae and yeast cells (Figure 4D). At 25°C, the colony surface of the Δ*pakB* strain resembled that of wildtype at 37°C (Figure 4E). The Δ*pakB* strain also appeared to produce more yeast cells than wildtype at 37°C (Figure 4E).

### Deletion of *pakB* affects nuclear index and branching but not septation or actin distribution

**Figure 5:**
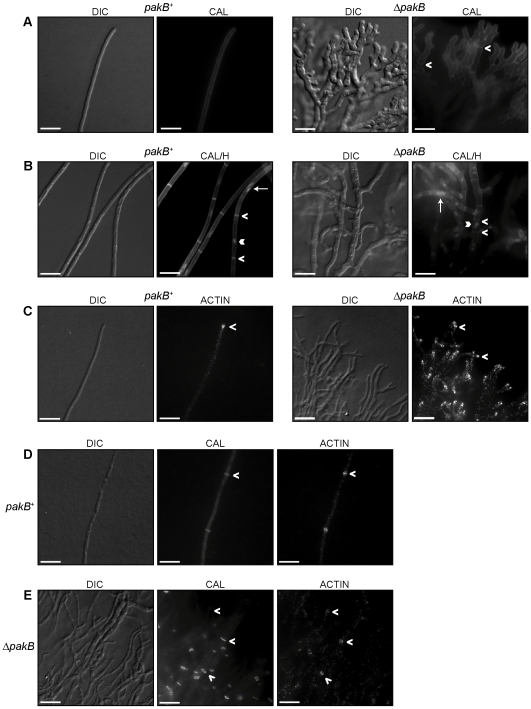
Hyphae of the Δ*pakB* strain show normal septation and actin distribution but have increased branching and nuclear index. Wildtype (*pakB* ^+^) and Δ*pakB* strains were grown on ANM + (NH_4_)_2_SO_4_ for 4 days at 25°C. (A) Branching does not occur in apical cells of wildtype (*pakB* ^+^) hyphae. The hyphae of the Δ*pakB* strain appear highly branched and frequent branches can be observed at the hyphal apex. Septa are visible in the Δ*pakB* strain (white arrowheads). (B) Co-staining with calcofluor and Hoechst 33258 reveals that wildtype (*pakB* ^+^) subapical cells are predominately uninucleate. Occasionally, dividing nuclei can be observed (white arrow). In contrast to wildtype, the subapical cells of the Δ*pakB* strain frequently possess 2 or more nuclei (white arrow). Cell ends indicated by white arrowheads and nuclei by double white arrowheads. (C) In wildtype (*pakB* ^+^) hyphae, actin is evident as cortical patches located along hyphae and is concentrated at the hyphal apex (white arrowhead). Both cortical actin patches and actin concentrated at the hyphal apex are observed in the Δ*pakB* strain (white arrowheads). (D & E) Actin is concentrated at nascent septation sites in wildtype (*pakB* ^+^) hyphae (white arrowhead). Like wildtype, actin in the Δ*pakB* strain is observed concentrated at nascent septation sites (white arrowheads). Images were captured using differential interference contrast (DIC) or with epifluorescence to observe calcofluor stained fungal cell walls (CAL), Hoechst 33258 stained nuclei (H) or immunofluorescently labeled actin (ACTIN). Scale bars, 20 µm.

To investigate further the molecular mechanisms underlying the deregulation of yeast cell morphogenesis in the Δ*pakB* strain at 25°C, the wildtype, Δ*pakB* and Δ*pakB pakB^+^* strains were grown for 4 days at 25°C, stained with calcofluor and Hoechst 33258 and observed microscopically (Figure 5). Wildtype *P. marneffei* grows as highly polarized, branched, septate hyphae (Figure 5). Apical cells are multinucleate whereas subapical cells are predominately uninucleate unless dividing (Figure 5B). In all conditions examined, the Δ*pakB pakB^+^* strains were indistinguishable from wildtype (data not shown). The Δ*pakB* strain exhibited a compact colony morphology where hyphae were tightly packed and highly branched (Figure 5A). Apical cell branching, which was not observed in wildtype, was frequently observed in the Δ*pakB* strain (Figure 5A). Septa were present in the Δ*pakB* strain (Figure 5A). In contrast to wildtype, the Δ*pakB* strain exhibited an increase in the number of nuclei per subapical cell compartment (Figure 5B). To quantify this increase, the number of nuclei per cellular compartment was recorded for 100 cells on three separate occasions. Only 20.5±2.04% and 20.4±2.72% of wildtype and Δ*pakB pakB^+^* subapical cells contained more than one nucleus, respectively. In contrast, 64.2±1.18% of subapical cells of the Δ*pakB* strain contained more than one nucleus.

To examine if deletion of *pakB* affects actin distribution, immunostaining using mouse anti-actin was performed on the wildtype and Δ*pakB* strains. In the wildtype at 25°C, actin is localized as cortical actin spots along the hyphae and concentrated at nascent septation sites and the hyphal apex (Figure 5C and D). Actin was normally distributed in the Δ*pakB* strain (Figure 5C and E).

### *pakB* is required for conidiation at 25°C and affects conidial germination kinetics

**Figure 6:**
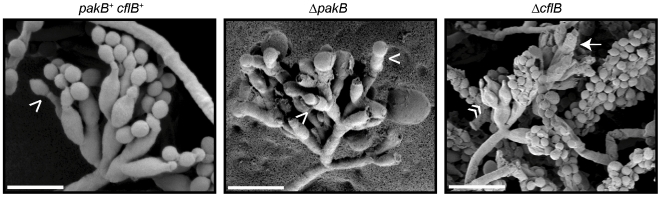
Deletion of *pakB* results in conidiation defects at 25°C. Wildtype (*pakB* ^+^ *cflB* ^+^), Δ*cflB* and Δ*pakB* strains were grown on ANM + (NH_4_)_2_SO_4_ for 14 days at 25°C. SEM of the surface of a wildtype (*pakB^+^ cflB^+^*) colony at 25°C shows numerous asexual development structures (conidiophores). In contrast to wildtype in which conidia are uniform in size, a large number of conidiophores of the Δ*pakB* strain bear abnormally large conidia. Single conidiophores can be observed in which separate phialides produce conidia which differ dramatically in size. More than one conidium per phialide is rarely observed and the site of conidium attachment to the phialide is not as constricted as in wildtype (white arrowheads). Like the Δ*pakB* strain, conidia of the Δ*cflB* strain can vary in size. Lysed conidiophore structures are frequently observed in the Δ*cflB* strain, in which the metulae and phialides have ruptured (white arrow) or deflated (double white arrowhead). Scale bars, 5 µm.

Wildtype *P. marneffei* begins asexual development after 4 days growth at 25°C, with the production of a specialized aerial stalk from which differentiated cells are produced sequentially in a budding fashion: metulae bud from the stalk, phialides bud from metulae and uninucleate conidia bud from phialides. To investigate if the deletion of *pakB* results in aberrant asexual development at 25°C, wildtype, Δ*pakB*, Δ*pakB pakB^+^* and Δ*cflB* strains were grown for 14 days at 25°C and were examined by SEM (Figure 6). Deletion of the *P. marneffei RAC* homologue, *cflB*, results in conidiation defects at 25°C [6]. Numerous conidiophores were observed on the surface of wildtype colonies (Figure 6). The Δ*pakB pakB^+^* strain was indistinguishable from the wildtype (data not shown). The Δ*pakB* strain also produced conidiophores in which all conidiophore cell types were observed, however a large number of conidiophores in the Δ*pakB* strain had abnormally large conidia (Figure 6). In addition, more than one conidium per phialide was rarely observed and the site of conidium to phialide attachment was not as constricted as in wildtype (Figure 6). In contrast to the wildtype, Δ*pakB* and Δ*pakB pakB^+^* strains, conidiophores in the Δ*cflB* strain could not be readily distinguished but presumptive conidia of varied size were noted, similar to the Δ*pakB* strain although not as extreme (Figure 6). A unique phenotype of the Δ*cflB* strain is that lysed conidiophore structures in which the metulae and phialides have ruptured or deflated were frequently observed (Figure 6).

**Figure 7:**
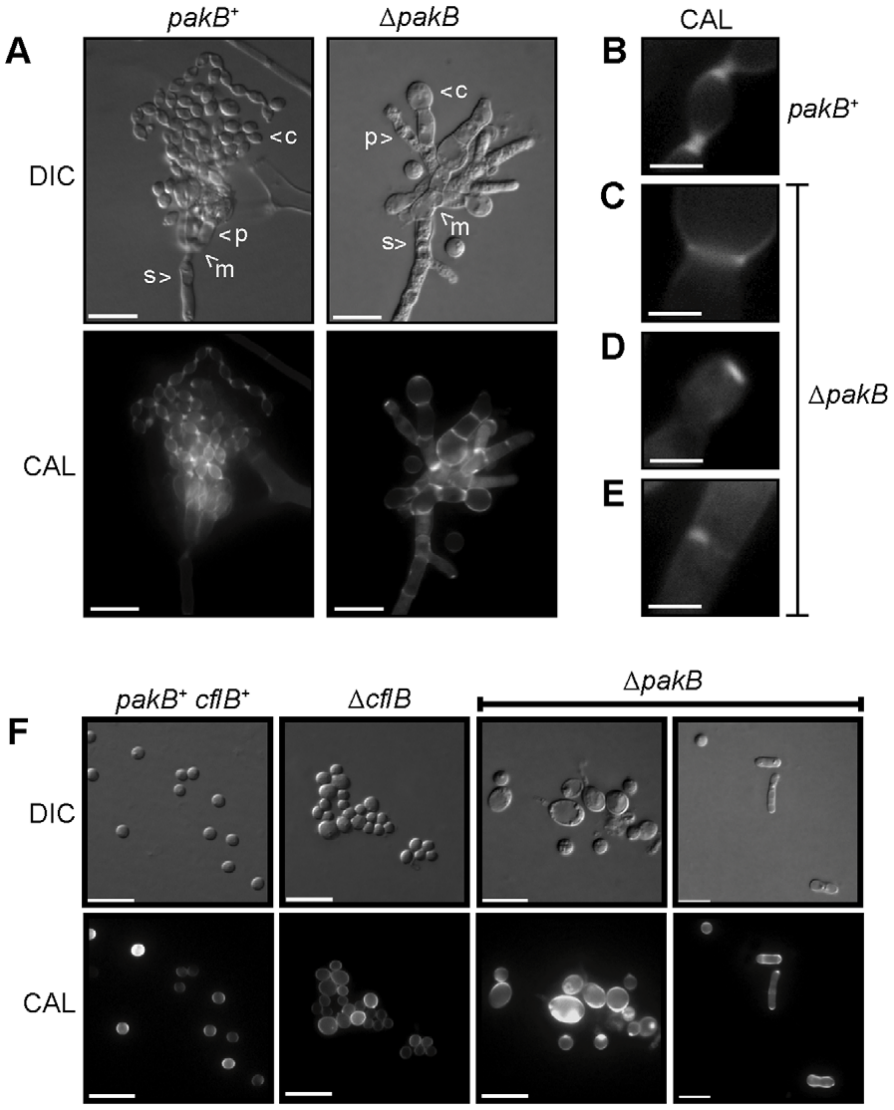
Δ*pakB* conidiophores display septation defects. Wildtype (*pakB* ^+^), Δ*cflB* and Δ*pakB* strains were grown on ANM + (NH_4_)_2_SO_4_ for 14 days at 25°C. (A) Conidiophores of wildtype (*pakB* ^+^) and Δ*pakB* strains. The conidiophores are comprised of a stalk (s), metulae (m), phialides (p) and chains of conidia (c). All cell types are observed in the Δ*pakB* strains. Conidia, and occasionally phialides, of Δ*pakB* conidiophores are misshapen and larger in size. Unlike the chains of conidia observed in wildtype conidiophores, more than one conidium per phialide is rarely observed in the Δ*pakB* conidiophores. (B–E) Septa in conidiophores. In wildtype conidiophores (B), two separate chitin disks can be observed at phialide to conidium and conidium to conidium connections, indicating cellular division and cell separation. This is not observed in the Δ*pakB* conidiophores (C–E). Only one septum (C), no septa (D) or incomplete septa (E) are observed at the phialide to conidia cell boundaries. (F) Conidia were scraped off the colony surface, re-suspended in 0.005% Tween 80 solution, filtered through Miracloth and stained with calcofluor (CAL). Conidia of wildtype (*pakB^+^ cflB^+^*) are uniform in size. In contrast, conidia of the Δ*cflB* strain varied in size. Similar to the Δ*cflB* strain, conidia of the Δ*pakB* strain also varied in size. However, conidia of the Δ*pakB* strain were swollen to a greater extent than those from the Δ*cflB* strain and often exhibited uneven calcofluor staining. In addition, numerous yeast cells were observed in the Δ*pakB* strain. Images were captured using differential interference contrast (DIC) or with epifluorescence to observe calcofluor stained fungal cell walls (CAL). Scale bars, 20 µm (A and F) and 2.5 µm (B–E).

To investigate the cellular defects underlying the aberrant conidiophores of the Δ*pakB* strain, calcofluor staining was performed on wildtype, Δ*pakB* and Δ*pakB pakB^+^* strains after 14 days growth at 25°C (Materials and Methods). The Δ*pakB* conidiophores displayed septation defects in conidiophores (Figure 7A–B). In contrast to wildtype conidiophores, in which two separate chitin disks can be observed at the phialide/conidium and conidium/conidium junctions, only one septum, no septa or incomplete septa were observed at the phialide to conidia cell boundaries in Δ*pakB* conidiophores (Figure 7B).

To assess any defects in conidia that may be the result of the aberrant conidiophore morphogenesis in the mutant strains, calcofluor staining was performed on conidial suspensions of the wildtype, Δ*pakB*, Δ*pakB pakB^+^* and Δ*cflB* strains (Materials and Methods). The conidia produced by the wildtype and Δ*pakB pakB^+^* strains were homogeneous in size and showed uniform calcofluor staining around the cell periphery (Figure 7C and data not shown). A proportion of conidia produced by the Δ*cflB* strain displayed a size increase, however, calcofluor staining remained uniform (Figure 7C). In contrast, conidia from the Δ*pakB* strain differed greatly in size and showed uneven calcofluor staining (Figure 7C). In addition, Δ*pakB* conidial preparations contained numerous yeast cells (Figure 7C). Nuclear staining revealed that these yeast cells were uninucleate (data not shown). The germination and colony forming ability of conidia from the wildtype, Δ*pakB*, and Δ*cflB* strains was determined to investigate the potential consequences of the aberrant morphogenesis. The germination kinetics were measured by counting the number of ungerminated versus germinated conidia (conidia with a visible germ tube) in a population of 100 in three independent experiments after 15 hours in liquid medium at both 25°C and 37°C (Table 1). Despite the large increase in conidial size, conidia of the Δ*pakB* strain germinated well at both 25°C and 37°C and actually showed a slight increase in germination compared to the wildtype control (Table 1). In contrast, the conidia of the Δ*cflB* strain showed a reduction in germination compared to the other strains (Table 1). It was also evident that Δ*pakB* conidia prematurely extended secondary germ tubes. To quantify this, the number of conidia with 1, 2 or 3 or more germ tubes was counted in a population of 100 in three independent experiments (Table 2). After 15 hrs at either 25°C or 37°C, the Δ*pakB* conidia showed a significant increase in germ tube emergence (Table 2). The ability of single conidia to form colonies was also assessed after 5 days at both 25°C and 37°C (Table 3). The colony forming units were measured by counting the number of colonies arising from 100 plated conidia in three independent experiments (Table 3). Consistent with the germination data, the Δ*pakB* strain showed wildtype viability (Table 3). The Δ*cflB* strain showed a reduction in the ability to form colonies suggesting that the previously observed ungerminated conidia were inviable rather than delayed (Table 3).

**Table 1:** % Germination of conidia after 15 hours at 25°C and 37°C.

Strain	% Germination at 25°C	% Germination at 37°C
*pakB^+^ cflB^+^*	84.2±3.52	75.6±4.90
Δ*pakB*	91.2±1.07	90.2±3.05
Δ*pakB pakB^+^*	85.3±5.47	73.9±3.37
Δ*cflB*	41.5±4.00	31.7±3.15

**Table 2:** % Conidia with multiple germ tubes after 15 hrs at 25°C or 37°C.

Temperature	Strain	% Conidia with 1 germ tube	% Conidia with 2 germ tubes	% Conidia with 3 or more germ tubes
25°C	*pakB^+^*	56.8±3.43	41.1±2.17	2.13±1.34
25°C	Δ*pakB*	11.0±3.24	63.5±4.31	25.5±4.54
25°C	Δ*pakB pakB^+^*	51.7±2.48	47.2±2.73	1.17±0.27
37°C	*pakB^+^*	54.2±8.21	41.4±8.03	4.33±0.79
37°C	Δ*pakB*	11.4±1.37	52.3±4.53	36.2±3.17
37°C	Δ*pakB pakB^+^*	51.5±0.93	45.5±1.76	2.97±0.84

**Table 3:** % Colony forming units after 5 days at 25°C and 37°C.

Strain	% Viability at 25°C	% Viability at 37°C
*pakB^+^ cflB^+^*	100±0.00	86.0±8.57
Δ*pakB*	100±0.00	91.2±5.38
Δ*pakB pakB^+^*	100±0.00	85.8±2.80
Δ*cflB*	55.3±8.74	48.3±3.33

### Yeast cells produced by the Δ*pakB* strain at 25°C are derived from conidiophores

When examining asexual development in the Δ*pakB* strain, a number of conidiophores were observed in which the individual cell types had become detached (data not shown). As the Δ*pakB* strain inappropriately produced yeast cells at 25°C (Figure 4C and 7C) we hypothesized that these yeast cells may arise from an inappropriate switch from conidiation to arthroconidiation. To investigate this possibility, a Δ*pakB* Δ*brlA* double mutant was generated (Materials and Methods). *brlA* encodes the primary regulator of asexual development which is necessary and sufficient for asexual development. The Δ*brlA* mutant produces aerial stalks but is unable to produce the various budded cell types of the conidiophore (Borneman and Andrianopoulos, unpublished). Wildtype, Δ*pakB*, Δ*brlA* and Δ*pakB* Δ*brlA* strains were grown on ANM + (NH_4_)_2_SO_4_ for 14 days at 25°C. Cell suspensions were made in 0.005% Tween 80 solution and filtered through Miracloth to remove hyphal cells. As expected, conidia but no yeast cells were observed in wildtype suspensions while the Δ*pakB* strain produced conidia of varying size and numerous yeast cells. No conidia or yeast cells were observed in the Δ*brlA* strain. Interestingly, no conidia or yeast cells were observed for the Δ*pakB* Δ*brlA* strains suggesting that the yeast cells observed in the Δ*pakB* strain arise from the conidiation program (data not shown).

RNA was isolated from wildtype and Δ*brlA* strains grown for 4 days on solid medium at 25°C to investigate if *brlA* is required for *pakB* expression during asexual development. Expression of *pakB* during asexual development was similar in both wildtype and the Δ*brlA* strains showing that *pakB* expression during conidiation is independent of *brlA* (data not shown).

### PakB is not required for yeast cell morphogenesis *in vitro*

**Figure 8:**
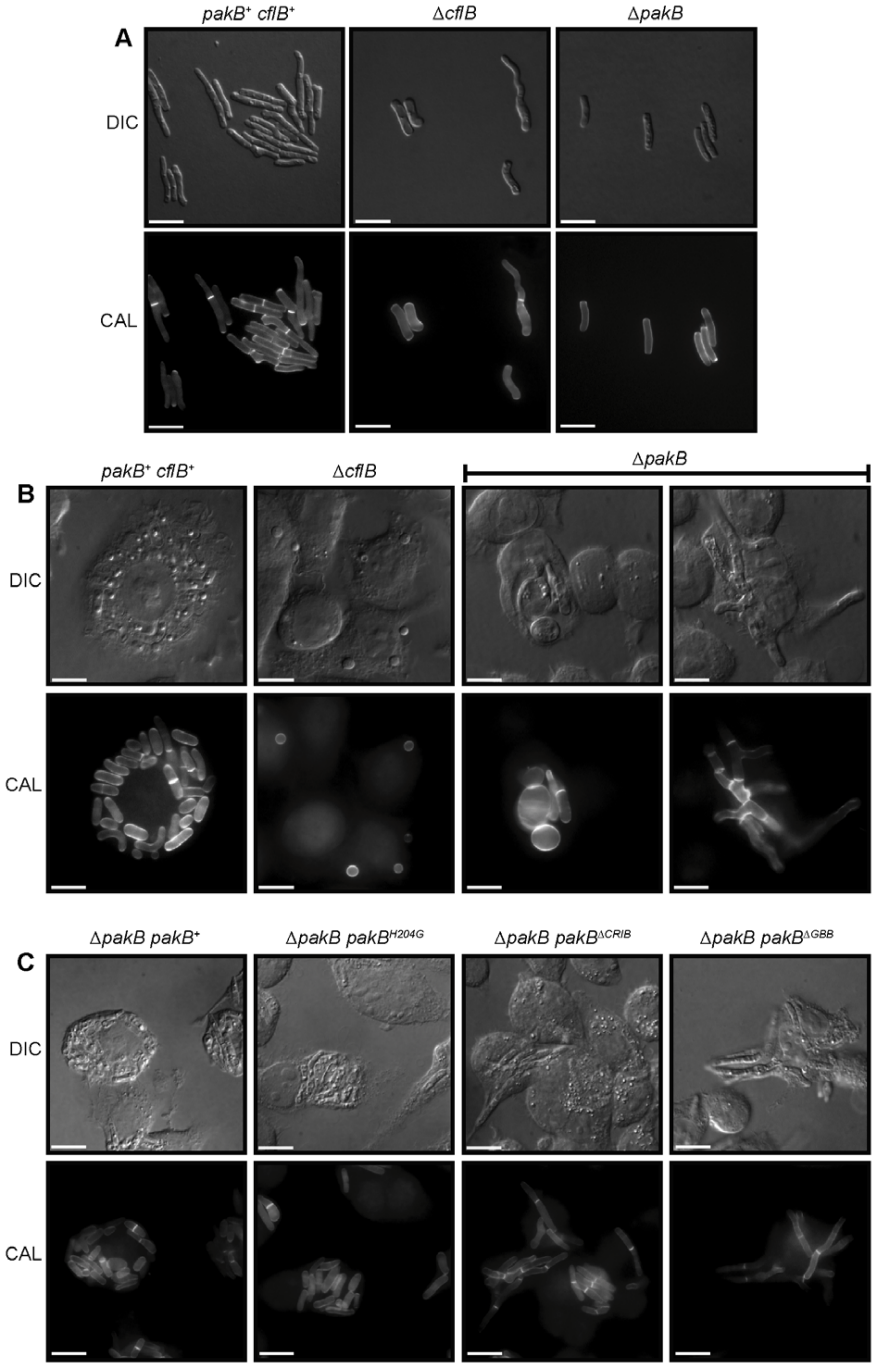
The Δ*pakB* strain displays morphological defects *in vivo*. (A) Wildtype (*pakB* ^+^ *cflB* ^+^), Δ*cflB* and Δ*pakB* strains were grown on BHI slides for 5 days at 37°C. Wildtype produces numerous yeast cells from arthroconidiating hyphae. Both the Δ*cflB* and Δ*pakB* strains produce numerous yeast cells from arthroconidiating hyphae, which are indistinguishable from wildtype. (B) LPS activated J774 murine macrophages infected with conidial suspensions of the wildtype (*pakB* ^+^ *cflB* ^+^), Δ*cflB* and Δ*pakB* strains. After 24 hours, numerous yeast cells dividing by fission were observed in macrophages infected with wildtype (*pakB* ^+^ *cflB* ^+^). In contrast, conidia of the Δ*cflB* strain remains predominately ungerminated in infected macrophages. Inside macrophages, some of the conidia of the Δ*pakB* strain appear large, swollen and ungerminated. However, the majority of infected macrophages contain highly branched, septate, hyphal Δ*pakB* cells. (C) LPS activated J774 murine macrophages infected with conidial suspensions of the Δ*pakB pakB* ^+^, Δ*pakB pakB^H204G^*, Δ*pakB pakB* ^Δ*CRIB*^ and Δ*pakB pakB* ^Δ*GBB*^ strains. After 24 hrs, the yeast cells produced by the Δ*pakB pakB* ^+^ and Δ*pakB pakB^H204G^* strains are indistinguishable from wildtype. Similar to the Δ*pakB* mutant, the yeast cells produced by both the Δ*pakB pakB* ^Δ*CRIB*^ and Δ*pakB pakB* ^Δ*GBB*^ strains *in vivo* are long and septate. Images were captured using differential interference contrast (DIC) or with epifluorescence to observe calcofluor stained fungal cell walls (CAL). Scale bars, 20 µm.

Given the inappropriate production of yeast cell at 25°C, the effects of *pakB* deletion on *in vitro* yeast cell morphogenesis were assessed. Wildtype *P. marneffei* produced compact, mucoid, yeast colonies after 5 days growth at 37°C *in vitro* and both the Δ*pakB* and Δ*pakB pakB^+^* strains were indistinguishable from wildtype (Figure 3C). To examine yeast cell morphogenesis *in vitro*, the wildtype (*pakB^+^*), Δ*pakB*, Δ*pakB pakB^+^* and Δ*cflB* strains were inoculated on agar-coated BHI slides and incubated for 5 days at 37°C. Wildtype conidia germinate at 37°C to produce polarized arthroconidiating hyphae, an intermediary cell type which is specific to *in vitro* yeast morphogenesis and which is not manifested in macrophages. Nuclear division and septation become coupled in arthroconidiating hyphae, double septa are laid down and fragmentation occurs along this plane to liberate uninucleate yeast cells which consequently divide by fission. Numerous yeast cells were observed for the wildtype strain after 5 days at 37°C (Figure 8A). Similarly, the Δ*pakB*, Δ*pakB pakB^+^* and Δ*cflB* strains produced abundant yeast cells comparable to wildtype and these yeast cells were also uninucleate (Figure 8A). Thus PakB appears to play no role *in vitro*. This is consistent with the expression data under this condition in which the *pakB* transcript was barely detectable (Figure 1A). The wildtype, Δ*pakB* and Δ*pakB pakB^+^* strains also produced numerous yeast cells after 6 days growth at 37°C on Sab and ME yeast medium, comparable to those produced on BHI (data not shown).

### Expression of *pakB* mutant alleles cannot rescue the Δ*pakB* phenotype at 25°C

**Figure 9:**
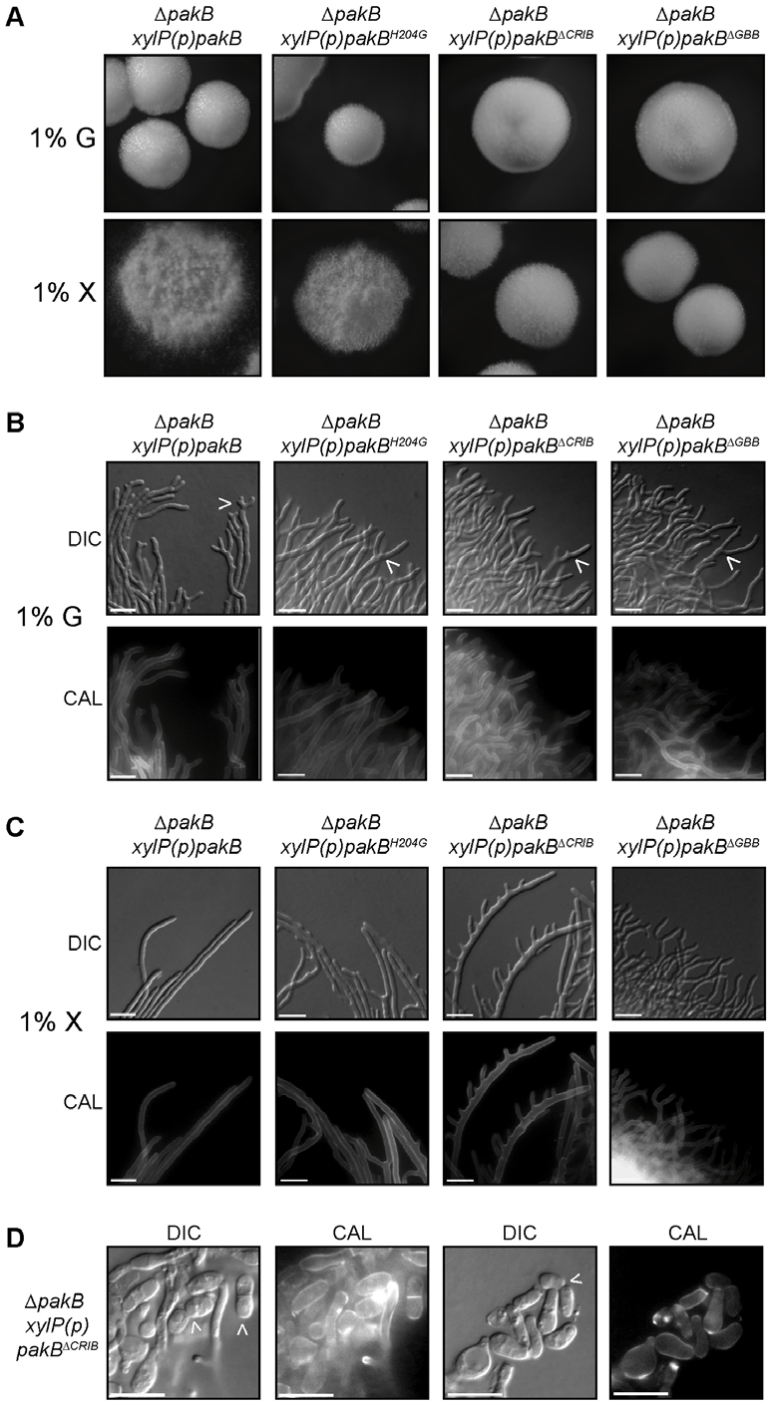
The CRIB and GBB domains are essential for PakB function at 25°C. Δ*pakB xylP(p)pakB*, Δ*pakB xylP(p)pakB^H204G^*, Δ*pakB xylP(p)pakB* ^Δ*CRIB*^ and Δ*pakB xylP(p)pakB* ^Δ*GBB*^ strains were grown on carbon-free ANM + (NH_4_)_2_SO_4_ supplemented with either 1% glucose or 1% xylose for 10 days (A) or 4 days (B–D) at 25°C. (A) All strains exhibit the Δ*pakB* yeast-like colonial phenotype at 25°C on non-inducing medium (1% G). On inducing medium (1% X), expression of the *xylP(p)pakB* construct completely restores the wildtype hyphal phenotype. Expression of the *xylP(p)pakB^H204G^* allele partially restores the wildtype hyphal phenotype. The Δ*pakB xylP(p)pakB* ^Δ*CRIB*^ and Δ*pakB xylP(p)pakB* ^Δ*GBB*^ strains are indistinguishable on non-inducing and inducing medium. (B) On non-inducing medium (1% G), all strains exhibit the Δ*pakB* phenotype at 25°C in that they produce compact colonies which are comprised of tightly packed, apically branched hyphae (indicated by white arrowheads). (C) On inducing medium (1% X), the expression of the *xylP(p)pakB* construct completely restores the wildtype hyphal phenotype. Expression of the *xylP(p)pakB^H204G^* allele partially restores the wildtype hyphal phenotype such that hyphae are less tightly packed and less apical branching is observed. Expression of the *xylP(p)pakB* ^Δ*CRIB*^ allele results in hyperbranching of hyphal cells. The Δ*pakB xylP(p)pakB* ^Δ*GBB*^ strains appear indistinguishable on 1% G and 1% X. (D) In addition to the hyphal hyperbranching observed in the Δ*pakB xylP(p)pakB* ^Δ*CRIB*^ strains on inducing medium (C), these strains produce numerous yeast cells around the colony periphery. Some yeast cells appear to be dividing by fission (white arrowheads). Images were captured using differential interference contrast (DIC) or with epifluorescence to observe calcofluor stained fungal cell walls (CAL). Scale bars, 20 µm.

In order to examine the role of conserved domains of PakB in the phenotypes observed, mutant alleles were generated which altered the CRIB (H204G and Δ195–256) and putative GBB domain (Δ719–729). The equivalent CRIB mutations in *S. cerevisiae* Ste20p prevents the Cdc42p interaction (H345G) or generate a constitutively active protein which bypasses the requirement for Cdc42p (ΔCRIB) [8]. Deletion of the GBB domain prevents Ste20p interaction with Ste4p, therefore affecting activation of the MAPK cascade [13]. These alleles, in addition to the wildtype allele, were placed under the control of the inducible *xylP* promoter, which is only expressed in the presence of xylose. The Δ*pakB pyrG^−^* strain was transformed with these constructs and transformants were directly selected for *pyrG^+^* and confirmed by Southern blot analysis of genomic DNA (Materials and Methods). The *xylP(p)pakB, xylP(p)pakB^H204G^, xylP(p)pakB* ^Δ*CRIB*^ and *xylP(p)pakB* ^Δ*GBB*^ strains were grown on media with or without 1% xylose for 10 days (Figure 9A) or for 4 days on agar-coated slides for microscopic observation (Figure 9B–D). As expected, all strains exhibited the Δ*pakB* phenotype at 25°C on non-inducing medium (Figure 9A–B). On inducing medium, expression of the *xylP(p)pakB* construct completely restored the wildtype phenotype (Figure 9A and C). Expression of the *xylP(p)pakB^H204G^* allele partially restored the wildtype hyphal phenotype such that colonies were more filamentous (Figure 9A) and hyphae were less tightly packed with less apical branching (Figure 9B–C). In contrast, strains expressing either the *xylP(p)pakB* ^Δ*CRIB*^ or *xylP(p)pakB* ^Δ*GBB*^ allele were indistinguishable at the colonial level on non-inducing and inducing medium (Figure 9A). Both the *xylP(p)pakB* ^Δ*CRIB*^ and *xylP(p)pakB* ^Δ*GBB*^ strains displayed compact, tightly packed hyphae exhibiting apical branching similar to Δ*pakB*, however, the *xylP(p)pakB* ^Δ*CRIB*^ strains were also hyperbranched and an increase in yeast cells around the colony periphery were observed (Figure 9C–D). These yeast cells appeared to be dividing by fission (Figure 9D).

To investigate the effect of the mutant alleles on asexual development at 25°C, these strains were grown for 14 days at 25°C and examined by SEM. All strains exhibited the Δ*pakB* phenotype on non-inducing medium. Unlike the Δ*pakB pakB^+^* strain, whose conidiophores were indistinguishable from wildtype, the *xylP(p)pakB* strain did not show full complementation on inducing medium; conidiophores mainly consisted of a single phialide and conidiophores with more than one conidium per phialide was rarely observed (data not shown). This suggests that the formation of complex multicellular conidiophores is sensitive to *pakB* expression levels (Figure 1A), either due to overexpression of PakB in conidiophore cell types or poor induction of the *xylP* promoter in conidiophore cell types. On inducing medium, the *xylP(p)pakB^H204G^, xylP(p)pakB* ^Δ*CRIB*^ and *xylP(p)pakB* ^Δ*GBB*^ strains were indistinguishable from the Δ*pakB* strain (data not shown).

To assess whether the H204G, ΔCRIB or ΔGBB mutations affect PakB localization, HA-tagged *pakB^H204G^*, *pakB* ^Δ*CRIB*^ and *pakB* ^Δ*GBB*^ constructs were generated and co-transformed with the *barA^+^* gene into the *P. marneffei* strain G487 (*niaD pyrG areA^−^*). Transformants were selected for glufosinate resistance and confirmed by Southern blot analysis of genomic DNA (Materials and Methods). Anti-HA immunostaining was performed on two strains of each genotype after 4 days growth at 25°C. Like wildtype, both the PakB^H204G^HA and PakB^ΔGBB^HA proteins were concentrated at the hyphal apex, however, the PakB^ΔCRIB^HA protein was not (data not shown). The PakB^H204G^HA, PakB^ΔGBB^HA and PakB^ΔCRIB^HA proteins showed wildtype localization in conidiophores and at nascent septation sites (data not shown).

### Expression of *pakB* mutant alleles at 37°C *in vitro* results in yeast cells which resemble those produced *in vivo*

**Figure 10:**
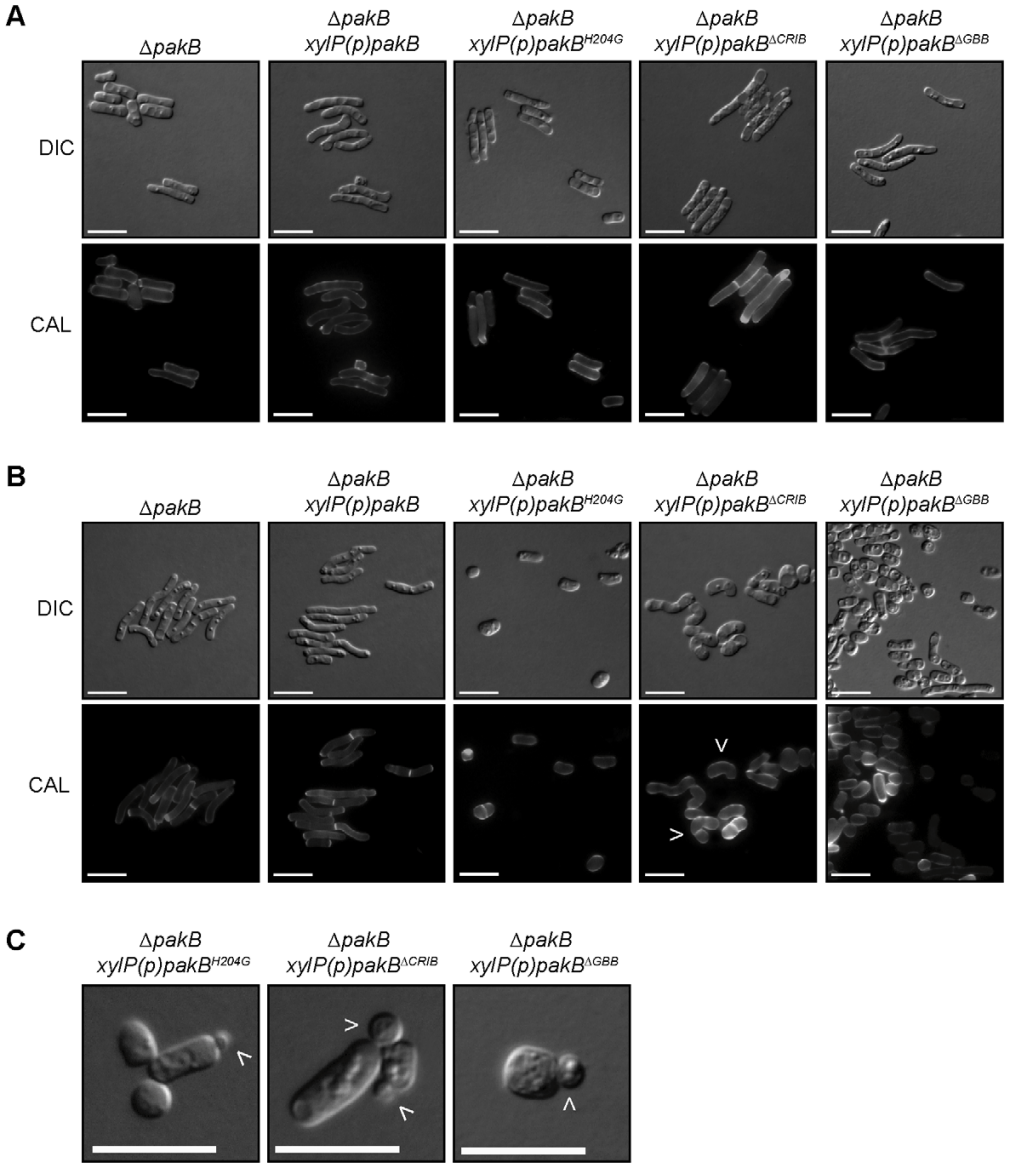
Expression of *pakB* mutant alleles at 37°C results in yeast cells resembling those produced *in vivo* but with budding division. Δ*pakB*, Δ*pakB xylP(p)pakB*, Δ*pakB xylP(p)pakB^H204G^*, Δ*pakB xylP(p)pakB* ^Δ*CRIB*^ and Δ*pakB xylP(p)pakB* ^Δ*GBB*^ strains were grown on BHI (A) or BHI + 1% xylose (B and C) for 5 days at 37°C. (A) Yeast cells produced by the Δ*pakB* exhibit wildtype morphology. The Δ*pakB xylP(p)pakB*, Δ*pakB xylP(p)pakB^H204G^*, Δ*pakB xylP(p)pakB* ^Δ*CRIB*^ and Δ*pakB xylP(p)pakB* ^Δ*GBB*^ strains are indistinguishable from the Δ*pakB* strain on non-inducing media. (B) Yeast cells produced by the Δ*pakB* and Δ*pakB xylP(p)pakB* strains on BHI +1% xylose exhibit wildtype morphology. The Δ*pakB xylP(p)pakB^H204G^*, Δ*pakB xylP(p)pakB* ^Δ*CRIB*^ and Δ*pakB xylP(p)pakB* ^Δ*GBB*^ strains produce yeast cells which are rounder and greatly reduced in length compared to both the Δ*pakB* and Δ*pakB xylP(p)pakB* strains. The yeast cells produced by the Δ*pakB xylP(p)pakB* ^Δ*CRIB*^ strains can also be curled (white arrowheads). (C) On inducing medium, a number of yeast cells produced by the Δ*pakB xylP(p)pakB^H204G^*, Δ*pakB xylP(p)pakB* ^Δ*CRIB*^ and Δ*pakB xylP(p)pakB* ^Δ*GBB*^ strains appear to be dividing in a budding-like manner (white arrowheads). Images were captured using differential interference contrast (DIC) or with epifluorescence to observe calcofluor stained fungal cell walls (CAL). Scale bars, 20 µm.

*pakB* was not expressed *in vitro* on BHI at 37°C (Figure 1A) but expression was induced during macrophage infection (Figure 1B). Yeast cells produced *in vivo* are shorter and rounder than those produced *in vitro* and develop directly from conidia rather than via arthroconidiating hyphae. To examine if induced expression of *pakB* can effect the changes in yeast cell morphology observed *in vivo*, the wildtype, Δ*pakB, xylP(p)pakB, xylP(p)pakB^H204G^, xylP(p)pakB* ^Δ*CRIB*^ and *xylP(p)pakB* ^Δ*GBB*^ strains were grown on BHI with or without 1% xylose for 5 days (Figure 10). As expected, all of the strains were indistinguishable from wildtype and the Δ*pakB* strain on non-inducing medium (Figure 10A). On inducing medium, the *xylP(p)pakB* strains (copy numbers 2–12) were indistinguishable from the wildtype and the Δ*pakB* strains (Figure 10B). Therefore an increase in *pakB* expression does not explain the production of rounded yeast cells *in vivo*. On inducing medium, the *xylP(p)pakB^H204G^, xylP(p)pakB* ^Δ*CRIB*^ and *xylP(p)pakB* ^Δ*GBB*^ strains produced yeast cells which were rounder and greatly reduced in length compared to both the Δ*pakB* and *xylP(p)pakB* strains and a number of yeast cells were produced which appeared to be dividing in a budding manner, similar to that observed during conidiation at 25°C (Figure 10B–C). The yeast cells produced by the *xylP(p)pakB* ^Δ*CRIB*^ strains were also often curled (Figure 10B).

### PakB is essential for yeast morphogenesis during infection

To investigate if *pakB* is required for yeast growth during infection, LPS activated J774 murine macrophages were infected with wildtype, Δ*pakB* and Δ*pakB pakB^+^* conidia and observed microscopically 24 hours post-infection (Materials and Methods). Calcofluor staining was performed to allow visualization of fungal cell walls. After 24 hours, numerous yeast cells dividing by fission were observed in macrophages infected with wildtype (*pakB^+^*) (Figure 8B) or Δ*pakB pakB^+^* conidia (data not shown). In contrast, the majority of macrophages infected with Δ*pakB* conidia contained highly branched, septate, hyphal cells but no yeast cells (Figure 8B). A small proportion of macrophages infected with Δ*pakB* conidia contained large, swollen conidia (Figure 8B). To quantify this difference, the number of cells containing at least one septum was recorded for approximately 100 cells on three separate occasions. In contrast to wildtype and Δ*pakB pakB^+^*, in which 17.2±3.70% and 15.3±1.74% of cells in infected macrophages contain septa, 71.1±2.24% of Δ*pakB* cells contain septa. This indicates that *pakB* is required for yeast cell division during infection. To determine if the role played by PakB *in vivo* is during the initiation or maintenance of yeast-like growth, macrophages were also infected with wildtype, Δ*pakB* and Δ*pakB pakB^+^* yeast cells (Materials and Methods). Similar to infection with conidia, after 24 hours numerous yeast cells were observed in macrophages infected with wildtype or Δ*pakB pakB^+^* yeast cells whereas macrophages infected with Δ*pakB* yeast cells contained only septate hyphal cells (Figure S2).

To assess if cellular morphology affects the oxidative state of the host, superoxide production was detected by Nitrotetrazolium Blue Chloride (NBT) staining of macrophages 24 hours post-infection (Materials and Methods). No difference in superoxide production was observed in macrophages infected with either wildtype or the Δ*pakB* strain (data not shown). In addition, the presence or absence of superoxide did not affect the growth phenotype of the Δ*pakB* strain (data not shown).

To examine whether the presence of host extracts is sufficient to induce the morphological switch, lysed macrophage extracts were added to wildtype, Δ*pakB* and Δ*pakB pakB^+^* conidia (Materials and Methods). After 24 hrs at 37°C, all strains were growing as hyphae indicating that the addition of lysed macrophage extracts is insufficient to induce yeast-like growth (data not shown). To observe if being intracellular is sufficient to induce the morphological switch, macrophages infected with wildtype, Δ*pakB* and Δ*pakB pakB^+^* conidia were incubated at 25°C. Unlike at 37°C, after 24 hrs all strains grew as hyphae indicating that it is a combination of host and temperature signals which induces yeast-like growth (data not shown).

To investigate if the mutant alleles can complement the phenotype of the Δ*pakB* strain *in vivo*, *pakB^+^, pakB^H204G^*, *pakB* ^Δ*CRIB*^ and *pakB* ^Δ*GBB*^ constructs were co-transformed with the *barA^+^* gene into the Δ*pakB* strain and transformants were selected for glufosinate resistance and confirmed by Southern blot analysis of genomic DNA (Materials and Methods). These strains exhibited identical phenotypes to the equivalent *xylP* overexpression strains grown on inducing medium at 25°C (data not shown). LPS activated J774 murine macrophages were infected with conidia from these strains and examined microscopically after 24 hours (Materials and Methods). Calcofluor staining was performed to allow visualization of fungal cell walls. The number of cells containing a septum was recorded for approximately 100 cells, in four transformants of each genotype, in three independent experiments (Table S1) and significant differences assessed by two-level nested analysis of variance (ANOVA) (Table S2). ANOVA showed there was no significant difference between the wildtype, Δ*pakB pakB^+^* and Δ*pakB pakB^H204G^* strains but there was a significant difference between these strains and the Δ*pakB*, Δ*pakB pakB* ^Δ*CRIB*^ and Δ*pakB pakB* ^Δ*GBB*^ strains. There was also a significant difference between the Δ*pakB pakB* ^Δ*CRIB*^ strains and either the Δ*pakB pakB* ^Δ*GBB*^ or Δ*pakB* strains. No significant differences were detected between transformants of the same genotype (Table S2). After 24 hours, numerous yeast cells dividing by fission were observed in macrophages infected with wildtype, Δ*pakB pakB^+^* or Δ*pakB pakB^H204G^* conidia (Figure 8C) of which 17.2±3.70% (wildtype), 19.5±0.86% (Δ*pakB pakB^+^*) and 22.2±1.3% (Δ*pakB pakB^H204G^*) of cells contained at least one septum. In contrast, macrophages infected with either of the Δ*pakB*, Δ*pakB pakB* ^Δ*CRIB*^ or Δ*pakB pakB* ^Δ*GBB*^ strains contained both septate yeast and hyphal cells (Figure 8C) and the number of septa was substantially higher than the wildtype (wildtype 17.2±3.70%; Δ*pakB* strain 71.1±2.24%; Δ*pakB pakB* ^Δ*CRIB*^ 42.6±1.9%; Δ*pakB pakB* ^Δ*GBB*^ 62.2±1.99%).

To assess whether the H204G, ΔCRIB or ΔGBB mutations affect PakB localization during macrophage infection, anti-HA immunostaining was performed on the PakB^H204G^HA, PakB^ΔCRIB^HA and PakB^ΔGBB^HA strains after 24 hours post-infection of LPS activated J774 murine macrophages (Materials and Methods). The PakB^H204G^HA, PakB^ΔGBB^HA and PakB^ΔCRIB^HA proteins showed wildtype localization during macrophage infection (data not shown).

The Rho type GTPases CDC42 and Rac are known regulators of PAKs and interact via the CRIB domain. As the Δ*pakB pakB^H204G^* and Δ*pakB pakB* ^Δ*CRIB*^ strains showed either no effect or only partial deregulation of yeast morphogenesis during infection, the *in vivo* phenotype of *cflA^G14V^*, *cflA^120A^* and Δ*cflB* mutants was assessed [5],[6] (Materials and Methods). After 24 hours, numerous yeast cells dividing by fission were observed in macrophages infected with wildtype (*pakB^+^*) (Figure 8B). In contrast, Δ*cflB* conidia in infected macrophages remained predominately ungerminated (Figure 8B) (76.2±6.00% for Δ*cflB* compared to 13.3±4.86% for wildtype). Unexpectedly, numerous yeast cells with wildtype morphology were observed in macrophages infected with the *cflA^G14V^* and *cflA^120A^* mutants (Figure S3). These results suggest that any interaction between CflA and PakB is non-essential for *in vivo* morphogenesis and that the defects in yeast cell morphology observed in the *cflA^G14V^* and *cflA^120A^* mutants *in vitro* [5] are circumvented when growing inside host cells.

## Discussion

### Intracellular morphogenesis and growth

Many pathogens reside within host phagocytic cells where they evade much of the host immune system. For dimorphic fungal pathogens a central attribute which facilitates this immune system avoidance is the capacity to switch from a multicellular hyphal growth form to a unicellular yeast form and it has been demonstrated that blocking this transition abrogates pathogenicity [15],[16]. Host body temperature (37°C) is the clearest *ex vivo* inducer of the hyphal to yeast transition in many dimorphic fungi and it is often assumed that this is the inducer *in vivo*. Here we show that *pakB*, which encodes the second p21 activated kinase in the dimorphic pathogen *P. marneffei*, is strongly upregulated upon phagocytosis by macrophages and is essential for yeast morphogenesis but not growth in macrophages. In contrast PakB plays no role in yeast morphogenesis at 37°C *in vitro*. This clearly places PakB in a new signalling pathway which responds to host cell inductive signals, not temperature, and is necessary for intracellular yeast morphogenesis and consequently pathogenicity.

The mechanism by which PakB controls yeast cell morphogenesis inside host cells is unique. The Rho type GTPases CDC42 and Rac are known regulators of PAKs in many eukaryotes, interacting via the CRIB domain of these kinases. Consistent with this PakB and CflB orthologues from a number of fungal pathogens have been shown to either physically or genetically interact, yet none of these systems represent intracellular pathogens. The *P. marneffei* Δ*cflB* (Rac) mutant strain fails to germinate in macrophages and mutation of the PakB CRIB domain (*pakB^H204G^*) does not recapitulate this phenotype nor that of the Δ*pakB* mutant. In strains where the entire CRIB or predicted Gβ binding domains are deleted, there is a partial deregulation of morphogenesis leading to the production of both yeast and hyphal cells in macrophages. Previous studies that have shown that CflA is required for correct yeast cell morphogenesis in *P. marneffei* during *in vitro* growth, potentially implicating CflA as a regulator for PakB. However the morphology of yeast cells in CRIB domain *pakB* mutants, both *in vitro* and inside macrophages, is essentially wildtype and *cflA^G14V^* and *cflA^120A^* mutants produce wildtype yeast cells *in vivo* showing that any interaction between CflA and PakB is non-essential for *in vivo* morphogenesis and that the defects in yeast cell morphogenesis observed in the *cflA^G14V^* and *cflA^120A^* mutants *in vitro* may be due to a general defects in morphogenesis.

In dimorphic fungi which have a predominant yeast phase such as *C. albicans* and *Y. lipolytica*, mutation of the *pakB* orthologue (*CLA4*) leads to defective hyphal formation and invasive growth, and consequent changes in pathogenicity for *C. albicans* [17],[18]. For fungi whose predominant growth phase is hyphal and pathogenic phase is yeast, mutation of *pakB* has the opposite effect. The simplest explanation for these opposing effects is that *pakB* orthologues control a conserved fundamental cellular process which is recruited by the organism-specific regulatory systems for either the yeast-hyphal or hyphal-yeast dimorphic switch. Based on the results described here, and discussed below, this process is likely to be the regulation of both morphogenesis and septation during cellular division.

### PakB is required for hyphal morphogenesis at 25°C

Dimorphic fungal pathogens must regulate both the transition and maintenance of two vegetative growth states, multicellular hyphal and unicellular yeast, as well as other morphogenetic programs such as asexual development [19]. This capacity is lost when the *pakB* gene is deleted from *P. marneffei*. The *pakB* gene is specifically expressed during hyphal growth and asexual development at 25°C and is required for the generation of these 25°C specific cell types. Loss of *pakB* leads to defects in polarised growth of hyphal cells and these defects partially overlap with those for *cflA* (*CDC42*) and *cflB* (*Rac*) mutants. In contrast, the second PAK in *P. marneffei* plays no role in hyphal cell morphogenesis [12]. Furthermore, loss of *pakB* leads to inappropriate production of yeast cells at 25°C and it might be argued that PakB is either required to promote hyphal growth or to negatively regulate yeast growth, and that yeast growth is the default state. In support of this hypothesis, a number of hyphal specific genes showed decreased expression while yeast specific genes showed increased expression in the Δ*pakB* strain. Studies of the *P. marneffei* transcriptional co-repressor *tupA* lead to a similar conclusion about the default growth state [20].

Whilst the nature of the default state is likely to be correct, it is clear that *pakB* plays a more fundamental role beyond hyphal morphogenesis. The inappropriately produced yeast cells in the *ΔpakB* strain are not derived from vegetative hyphal cells but from differentiating conidiophore cells. Deletion of the primary regulator of asexual development which is not expressed in vegetative hyphae, the C_2_H_2_ Zn finger transcription factor gene *brlA*, abolished production of yeast cells at 25°C in the Δ*pakB* strain. This suggests overlap in the morphogenetic mechanisms controlling conidiation at 25°C and yeast cell production at 37°C and supports previous studies in *P. marneffei* and other dimorphic fungi [16],[20],[21],[22],[23]. More importantly it points to the underlying mechanism being the control of the mode of cellular division. One of the major differences between vegetative hyphal cells and differentiated conidiophore cells is that the former divide by septation (analogous to fission but without cell separation) while the latter divide by budding, so a possible explanation is that PakB is required for the correct execution of budding division during conidiation in *P. marneffei* and loss of PakB defaults to a fission mode of division with cell separation in conidiophore cell types; as occurs in *P. marneffei* yeast cells at 37°C. In support of this hypothesis, deletion of the *CLA4* homologue in *U. maydis* results in yeast cells which separate by fission, instead of the normal budding mode at the distal tips of yeast cells, with constriction occurring at a centrally located septum [24]. Although this situation differs from *P. marneffei*, as it is occurring in undifferentiated cell types, it suggests that fission is acting as the default mechanism for division when budding is aberrant. These results suggest that budding is a derived mode of division and this is consistent with our understanding of the molecular mechanisms which underlie budding, as septation (fission) follows the isotropic growth phase during the budding program.

### *pakB* regulates septation during cellular division

Asexual development is a developmental program in which the hyphal-based apical growth and septation mode of growth switches to acropetal budding division without cell separation and finally basipetal budding division with cell separation, with the concomitant switch to uninucleate cells. Many dimorphic fungi produce yeast cells that divide by budding while *P. marneffei* produces yeast cells that divide by fission. In all of these instances, the coupling of septation to cell separation determines whether yeast or hyphal (or pseudohyphal) cells will be generated. The *pakB* deletion strain is able to undergo asexual development, albeit less profusely than wildtype, producing swollen conidia with an enlarged conidium attachment site, more akin to the septa of vegetative hyphal cells. The septa separating the other conidiophore cell types are also aberrant. This suggests that PakB activity regulates the constriction, and possibly formation, of septa during conidiogenesis and is supported by the localization of PakB to conidiophores where it is particularly concentrated at phialide to conidium boundaries. Despite the abnormal morphology of the Δ*pakB* conidia, they were still able to germinate normally at both 25°C and 37°C suggesting that it is only the final stages of conidial separation which are affected in the Δ*pakB* mutant. This is similar to the rice pathogen *Magnaporthe grisea* in which deletion of the *CLA4* homologue, *CHM1*, results in irregularly shaped conidia with reduced constriction at the conidium attachment site but is unlike the rye pathogen *Claviceps purpurea*, in which the Δ*cla4* strain is completely unable to sporulate [25],[26].

Loss of *pakB* completely blocks the link between septation and cell separation in *P. marneffei* during intracellular growth, leading to the formation of hyphal cells. This is consistent with the localization of PakB to septation sites *in vivo* where it is specifically localized to septa only after cell wall deposition had occurred suggesting that PakB is required for cell separation rather than septation. The Δ*pakB* phenotypes are consistent with the role of *CLA4* homologues in regulating cytokinesis. *S. cerevisiae*, *C. albicans* and *U. maydis CLA4* mutants are also unable to undergo normal cytokinesis during budding [18],[24],[27],[28]. In *S. cerevisiae*, *CLA4* is known to regulate the activity of Lte1p by localisation to the bud cortex, an important event in the Mitotic Exit Network (MEN), and loss of Lte1p leads to delayed cytokinesis [29],[30]. *P. marneffei* and *U. maydis* do not have clear *LTE1* orthologues and loss of *pakB* resulted in increased numbers of nuclei per cell compartment inconsistent with reduced or delayed mitotic exit. Furthermore, given the role of *CLA4* orthologues in cytokinesis, it may have been expected that deletion of *P. marneffei pakB* would also result in septation defects in vegetative cells at 25°C. However, Δ*pakB* hyphae displayed normal calcofluor stained septa and actin ring formation. Normal calcofluor-white stained septa were also present in the *CLA4* deletion strains of *C. purpurea, M. grisea* and *A. gossypii* [25],[26],[31]. Interestingly, the Δ*Agcla4* mutation in *A. gossypii* results in the absence of almost all actin rings [31].

### A role for PakB in the polarisome

The localization of wildtype PakB as a cap at the hyphal apex is very similar to that observed for the *A. nidulans* polarisome component SpaA [32]. The polarisome is a complex of polarity determining proteins originally identified in *S. cerevisiae* which include Bni1p (formin), Spa2p (scaffold), Bud6p and Pea2p. In *S. cerevisiae* Ste20p directly phosphorylates Bni1p [33]. Similar to the Δ*pakB* mutant, deletion of *A. nidulans sepA* (*BNI1*), *spaA* (*SPA2*) or *budA* (*BUD6*) results in apical branching [32],[34]. In contrast, deletion of *P. marneffei pakA* (*STE20*) does not result in apical branching and PakA co-localizes with actin to discrete spots concentrated at the hyphal apex which is inconsistent with polarisome morphology [12]. Thus, in contrast to *S. cerevisiae*, it is the Cla4p homologue PakB and not the Ste20p homologue PakA which activates the polarisome during hyphal growth.

The CRIB domain is required for Ste20p localization via interaction with Cdc42p in *S. cerevisiae*. Both the *STE20^ΔCRIB^* and *STE20^H345G^* mutations result in reduced localization to sites of polarized growth [8] as does the equivalent mutation (*pakA^H108G^*) in *P. marneffei* [12]. In contrast, the PakB^H204G^HA and PakB^ΔCRIB^HA proteins exhibited wildtype localization, with the exception of reduced localization to the hyphal apex in the latter, suggesting that the CRIB domain is largely non-essential for localization. This may represent a new paradigm for PAK function in non-yeast fungi and the mechanism by which PakB is correctly localised remains to be determined.

### p21-activated kinases control cellular morphogenesis and pathogenicity

*P. marneffei*, like many other dimorphic fungal pathogens, has two p21-activated kinases which are responsible for a variety of signaling and morphogenetic activities. The *pakA* gene encodes a Ste20p-like PAK which is essential for polarity establishment during conidial germination and polarised growth of the pathogenic yeast cells at 37°C such that conidia from *ΔpakA* strains fail to germinate upon phagocytosis by macrophages [12]. Based on genetic interaction studies it was shown that PakA lies downstream of CflA. However, *pakA* is not required for germination, polarised hyphal growth or asexual development at 25°C and it was postulated that this role may be filled by *pakB* [12]. Based on the data presented here, it is clear that *pakB* fulfils the predicted role of a polarity determinant during hyphal growth and asexual development but plays no role in conidial germination at 25°C, suggesting that this process, unlike its counterpart at 37°C and *in vivo*, may be PAK-independent. Formal proof of this hypothesis will require the generation of a Δ*pakA* Δ*pakB* double mutant. Unexpectedly, *pakB* plays a critical role in the formation of yeast cells in host cells, instead producing highly branched, septate, hyphal cells, but not *in vitro*. Upregulation of *pakB* expression in *P. marneffei* isolated from macrophages as opposed to *in vitro* cultured yeast cells shows that PakB activity is likely to be regulated at both the expression level as well as post-translationally. Identifying these host cell specific signals is the important next step in understanding how pathogens sense and respond to their hosts.

## Materials and Methods

### Molecular techniques

*P. marneffei* genomic DNA was isolated as previously described [21]. Southern and northern blotting was performed with Amersham Hybond N+ membrane according to the manufacturer's instructions. Filters were hybridized using [α-^32^P]dATP labeled probes by standard methods [35].

### Expression analysis

RNA was isolated from 2161 (wildtype) vegetative hyphal cells grown at 25°C for 2 days in liquid medium, from asexually developing cultures grown on solid medium at 25°C for 4 days and from yeast cells grown at 37°C for 6 days in liquid medium. RNA was isolated from yeast cells derived either from LPS activated J774 murine macrophages at 37°C infected with wildtype conidia 24 hours post-infection or from yeast cells incubated in macrophage growth medium (complete DMEM) at 37°C for 24 hours. Macrophages were infected as described below. RNA was also isolated from the Δ*pakB* strain after 2 days growth at 25°C in liquid medium and after 6 days at 37°C in liquid medium. RNA was extracted using TRIzol Reagent (Invitrogen) and a MP FastPrep-24 bead beater according to the manufacturer's instructions. RNA was DNaseI treated (Promega) prior to RT PCR analysis and a no cDNA synthesis control was performed to ensure no DNA contamination was present. Expression of *pakB, brlA* and the 25°C or 37°C specific probes 2E11, 2B10 and 2E4 was determined by RT PCR (Invitrogen Superscript III One-Step RT-PCR with Platinum Taq) using the primers: *pakB*-Y41 (5′- ACGGTGCGGTCGGAAAGA-3′), *pakB*-Y42 (5′- CTCCTTACGCACGGGCTG-3′), *brlA-*FF6 (5′- CATTCCCACAACCGATGACT-3′), *brlA-*FF7 (5′-CATACCTGGCGAGATCCACT-3′), 2E11-DD1 (5′-TTATTGTTGGCATTGGCG-3′), 2E11-DD2 (5′-TTATTGTTGGCATTGGCG-3′), 2B10-CC53 (5′-CGGTGCCGTACACAGGTATT-3′), 2B10-CC54 (5′-TTGATTTCAGGGCGGAGTAG-3′), 2E4-DD3 (5′-ATCCATCCCCCGTGAAGC-3′), 2E4-DD4 (5′-GCCGACACGAAGTGATCC-3′), *benA*-F58 (5′-GCTCCGGTGTCTACAATGGC-3′) and *benA*-F59 (5′-AGTTGTTACCAGCACCGGAC-3′). A range of cycle numbers was used to ensure the amplification was in the exponential phase and *benA* was used as an input RNA control.

### Cloning and plasmid construction

Previously, the *A. nidulans STE20* homologous sequence was used to screen a *P. marneffei* genomic library (constructed in λGEM-11) at low stringency (50% formamide, 2xSSC, 37°C) [12]. A 4 kb *SacII/XhoI* hybridizing fragment from a second positively hybridizing clone was subcloned into *SacII/XhoI* digested pBluescript II SK^+^ (pKB4752). This clone did not contain the entire *pakB* ORF so a 5.8 kb *PstI* hybridizing fragment was also subcloned into *PstI* digested pBluescript II SK^+^ (pKB5794). To generate a clone containing the entire *pakB* ORF, a 3.2 kb *SacI* fragment from pKB4752 was cloned into *SacI* digested pKB5794, this generated a 8.6 kb *pakB* clone (pKB4904). Double stranded sequencing was performed on 4.2 kb of clone pKB4904 and analyzed using Sequencher^TM^ 3.1.1 (Gene Codes Corporation).

A *pakB* deletion construct (pKB6019) was generated by cloning a 1.8 kb *PstI* fragment from a 2.9 kb *HindIII* SK^+^ subclone (pKB5746) into *PstI* digested pAB4626 (*pyrG^+^*), followed by cloning a 3 kb *BglII/XbaI* fragment from pKB4904 into *BamHI/XbaI* (generating pKB6019). This resulted in *pyrG^+^* flanked by 3.2 kb of 5′ and 1.8 kb of 3′ *pakB* sequence, and deleted from +323 to +2618.

To introduce the H204G mutation into *pakB*, inverse PCR using the mutagenic primers N64 (5′- GTCGGTTTCGATCCCAAGACT-3′) and N65 (5′- GTGGACACGACCGCTGAAATTGG-3′) was performed on a 4 kb *BgllII/StuI pakB* pLitmus 29 subclone (pKB5964). To introduce the ΔCRIB mutation, inverse PCR using the mutagenic primers S53 (5′- CGGGATCCCATTCCTGGGCACCGTTCGT-3′) and S54 (5′- CGGGATCCATGCGGGAACAGAACCCTCA-3′) was performed on pKB5964. This deletes from +595 to +783 of *pakB* (amino acids 195-256). The ΔGBB mutation was introduced by inverse PCR using the mutagenic primers AA7 (5′- AATGGAGGACAGTAAAAAGCC-3′) and AA8 (5′- TCGACTACAGCCCATTTTCA-3′) on pKB5964. This mutation deletes from +2628 to +2661 of *pakB* (amino acids 719-729). The promoter was added to these constructs by cloning a 1.8 kb *NcoI/BgllII* fragment into the *NcoI/BgllII* sites, generating pKB7306, pKB7171 and pKB6932, respectively. The integrity of the constructs was confirmed by sequencing.

The *xylP(p)pakB^+^ pyrG^+^* (pKB7024), *xylP(p)pakB^H204G^ pyrG^+^* (pKB7025), *xylP(p)pakB^ΔCRIB^ pyrG^+^* (pKB7026) and *xylP(p)pakB^ΔGBB^ pyrG^+^* (pKB7027) constructs were generated by PCR using the primers S51 (5′- CGTCTAGATGAACCCTGGACCAGCCCCG-3′) and S52 (5′- CCATCGATTACTGTCCTCCATTCTTCTT-3′), on pKB6941 (*xylP(p)pakB^+^ pyrG^+^*), pKB7306 (*xylP(p)pakB^HG^ pyrG^+^*), pKB7171 (*xylP(p)pakB^ΔCRIB^ pyrG^+^*) and pKB6932 (*xylP(p)pakB^ΔGBB^ pyrG^+^*), digesting the PCR product with *XbaI/ClaI* and cloning into *XbaI/ClaI* pDAP2 (*xylP(p) pyrG^+^*).

The HA tagged constructs were generated by replacing the 406 bp *SacII/HindII* fragment of *pakB* with the *SacII/HindII* fragment of pKB4693 (3x HA tag) in plasmids pKB6941, pKB7306, pKB7171 and pKB6932, generating pKB7116 (*pakB^+^* HA), pKB7118 (*pakB^H204G^* HA), pKB7172 (*pakB^ΔCRIB^* HA), and pKB7064 (*pakB^ΔGBB^* HA). This deletes from amino acid 328–343. This region is a poorly conserved region between the conserved CRIB and kinase domains and includes the first intron. To test for functionality, the pKB7116 (*pakB^+^* HA) plasmid was co-transformed with the *barA^+^* gene into the Δ*pakB* strain. Transformants were selected for glufosinate resistance and confirmed by Southern blot analysis of genomic DNA. Transformation of the Δ*pakB* strain with the *pakB^+^* HA plasmid (pKB7116) complemented the Δ*pakB* phenotype.

### Fungal strains and media

Strains used in this study are shown in Table 4. *P. marneffei* FRR2161, *cflA^G14V^*, *cflA^120A^* and Δ*cflB* have been previously described [5],[6]. Transformation was performed using the previously described protoplast method [21]. The Δ*pakB* strain (Δ*pakB::pyrG^+^*) was generated by transformation of strain G487 (*niaD^−^ pyrG^−^ areA^−^*) with linearized pKB6019 and selecting for pyrG^+^. The Δ*pakB pyrG^−^* strain was isolated by plating the Δ*pakB* strain (Δ*pakB::pyrG^+^*) on medium containing 1 mg mL^−1^ 5-fluoroorotic acid (5-FOA) supplemented with 10 mM γ-amino butyric acid (GABA) and 5 mM uracil to select for the loss of the *pyrG* marker. The strain is unable to grow in the absence of 5 mM uracil.

**Table 4:** *P. marneffei* strains used in this study.

Strain Name	Genotype	Copy # a
FR2161 (*pakB^+^)*	wildtype	NA
SPM4 *areA^−^*	*niaD1 pyrG1 areA^−^*	NA
Δ*pakB*	Δ*pakB::pyrG1^+^ niaD1 areA^−^*	NA
Δ*pakB pyrG^−^*	Δ*pakB pyrG1 niaD1 areA^−^*	NA
Δ*pakB pakB^+^*	Δ*pakB pyrG1 niaD1 areA^−^* [*pyrG1^+^*][*pakB^+^*]	2
Δ*pkuA pyrG^−^*	Δ*pkuA niaD1 pyrG1 areA^−^*	NA
Δ*brlA*	Δ*brlA::pyrG^+^* Δ*pkuA niaD1 pyrG1 areA^−^*	NA
Δ*brlA pyrG^−^*	Δ*brlA* Δ*pkuA niaD1 pyrG1 areA^−^*	NA
Δ*brlA* Δ*pakB*	Δ*brlA* Δ*pakB::pyrG^+^* Δ*pkuA niaD1 pyrG1 areA^−^*	NA
Δ*pakB pakB^+^* 2.1	Δ*pakB::pyrG1^+^ niaD1 areA^−^* [*barA^+^*][*pakB^+^*]	∼10
Δ*pakB pakB^+^* 2.2	Δ*pakB::pyrG1^+^ niaD1 areA^−^* [*barA^+^*][*pakB^+^*]	4
Δ*pakB pakB^+^* 2.3	Δ*pakB::pyrG1^+^ niaD1 areA^−^* [*barA^+^*][*pakB^+^*]	8
Δ*pakB pakB^+^* 2.4	Δ*pakB::pyrG1^+^ niaD1 areA^−^* [*barA^+^*][*pakB^+^*]	9
Δ*pakB pakB^H204G^* 1.1	Δ*pakB::pyrG1^+^ niaD1 areA^−^* [*barA^+^*][*pakA^H204G^*]	∼10
Δ*pakB pakB^H204G^* 1.3	Δ*pakB::pyrG1^+^ niaD1 areA^−^* [*barA^+^*][*pakA^H204G^*]	1
Δ*pakB pakB^H204G^* 1.4	Δ*pakB::pyrG1^+^ niaD1 areA^−^* [*barA^+^*][*pakA^H204G^*]	8
Δ*pakB pakB^H204G^* 2.4	Δ*pakB::pyrG1^+^ niaD1 areA^−^* [*barA^+^*][*pakA^H204G^*]	6
Δ*pakB pakB* ^Δ*CRIB*^ 2.1	Δ*pakB::pyrG1^+^ niaD1 areA^−^* [*barA^+^*][*pakA* ^Δ*CRIB*^]	∼15
Δ*pakB pakB* ^Δ*CRIB*^ 2.3	Δ*pakB::pyrG1^+^ niaD1 areA^−^* [*barA^+^*][*pakA* ^Δ*CRIB*^]	8
Δ*pakB pakB* ^Δ*CRIB*^ 2.4	Δ*pakB::pyrG1^+^ niaD1 areA^−^* [*barA^+^*][*pakA* ^Δ*CRIB*^]	10
Δ*pakB pakB* ^Δ*CRIB*^ 2.5	Δ*pakB::pyrG1^+^ niaD1 areA^−^* [*barA^+^*][*pakA* ^Δ*CRIB*^]	9
Δ*pakB pakB* ^Δ*BBD*^ 2.1	Δ*pakB::pyrG1^+^ niaD1 areA^−^* [*barA^+^*][*pakA* ^Δ*GBB*^]	2
Δ*pakB pakB* ^Δ*BBD*^ 2.2	Δ*pakB::pyrG1^+^ niaD1 areA^−^* [*barA^+^*][*pakA* ^Δ*GBB*^]	13
Δ*pakB pakB* ^Δ*BBD*^ 2.4	Δ*pakB::pyrG1^+^ niaD1 areA^−^* [*barA^+^*][*pakA* ^Δ*GBB*^]	11
Δ*pakB pakB* ^Δ*BBD*^ 2.6	Δ*pakB::pyrG1^+^ niaD1 areA^−^* [*barA^+^*][*pakA* ^Δ*GBB*^]	∼15
Δ*pakB xylP(p)pakB^+^* 1.1	Δ*pakB pyrG1 niaD1 areA^−^* [*xylP(p)pakB^+^pyrG1^+^*]	7
Δ*pakB xylP(p)pakB^+^* 1.2	Δ*pakB pyrG1 niaD1 areA^−^* [*xylP(p)pakB^+^pyrG1^+^*]	12
Δ*pakB xylP(p)pakB^+^* 2.2	Δ*pakB pyrG1 niaD1 areA^−^* [*xylP(p)pakB^+^pyrG1^+^*]	2
Δ*pakB xylP(p)pakB^+^* 2.6	Δ*pakB pyrG1 niaD1 areA^−^* [*xylP(p)pakB^+^pyrG1^+^*]	7
Δ*pakB xylP(p)pakB^H204G^* 1.3	Δ*pakB pyrG1 niaD1 areA^−^* [*xylP(p)pakB^H204G^ pyrG1^+^*]	13
Δ*pakB xylP(p)pakB^H204G^* 1.5	Δ*pakB pyrG1 niaD1 areA^−^* [*xylP(p)pakB^H204G^ pyrG1^+^*]	8
Δ*pakB xylP(p)pakB^H204G^* 2.13	Δ*pakB pyrG1 niaD1 areA^−^* [*xylP(p)pakB^H204G^ pyrG1^+^*]	2
Δ*pakB xylP(p)pakB^H204G^* 2.16	Δ*pakB pyrG1 niaD1 areA^−^* [*xylP(p)pakB^H204G^ pyrG1^+^*]	3
Δ*pakB xylP(p)pakB^ΔCRIB^* 1.2	Δ*pakB pyrG1 niaD1 areA^−^* [*xylP(p) pakB^ΔCRIB^ pyrG1^+^*]	12
Δ*pakB xylP(p)pakB^ΔCRIB^* 2.1	Δ*pakB pyrG1 niaD1 areA^−^* [*xylP(p) pakB^ΔCRIB^ pyrG1^+^*]	3
Δ*pakB xylP(p)pakB^ΔCRIB^* 2.4	Δ*pakB pyrG1 niaD1 areA^−^* [*xylP(p) pakB^ΔCRIB^ pyrG1^+^*]	5
Δ*pakB xylP(p)pakB^ΔCRIB^* 2.5	Δ*pakB pyrG1 niaD1 areA^−^* [*xylP(p) pakB^ΔCRIB^ pyrG1^+^*]	2
Δ*pakB xylP(p)pakB^ΔGBB^* 1.2	Δ*pakB pyrG1 niaD1 areA^−^* [*xylP(p) pakB^ΔGBB^ pyrG1^+^*]	15
Δ*pakB xylP(p)pakB^ΔGBB^* 1.3	Δ*pakB pyrG1 niaD1 areA^−^* [*xylP(p) pakB^ΔGBB^ pyrG1^+^*]	13
Δ*pakB xylP(p)pakB^ΔGBB^* 2.8	Δ*pakB pyrG1 niaD1 areA^−^* [*xylP(p) pakB^ΔGBB^ pyrG1^+^*]	2
Δ*pakB xylP(p)pakB^ΔGBB^* 2.9	Δ*pakB pyrG1 niaD1 areA^−^* [*xylP(p) pakB^ΔGBB^ pyrG1^+^*]	9
Δ*cflB*	Δ*cflB::pyrG1^+^ niaD1*	NA
*cflA^G14V^*	*pyrG1 niaD1* [*cflA^G14V^*][*pyrG1^+^*]	4–8
*cflA^D120A^*	*pyrG1 niaD1* [*cflA^D120A^*][*pyrG1^+^*]	4–8
*pakB^+^* HA 1.4	*niaD1 pyrG1 areA^−^* [*barA^+^*][*pakB^+^* HA]	6
*pakB^+^* HA 1.6	*niaD1 pyrG1 areA^−^* [*barA^+^*][*pakB^+^* HA]	2
*pakB^HG^* HA 1.1	*niaD1 pyrG1 areA^−^* [*barA^+^*][*pakB^HG^* HA]	9
*pakB^HG^* HA 1.2	*niaD1 pyrG1 areA^−^* [*barA^+^*][*pakB^HG^* HA]	10
*pakB^ΔCRIB^* HA 1.2	*niaD1 pyrG1 areA^−^* [*barA^+^*][*pakB^ΔCRIB^* HA]	5
*pakB^ΔCRIB^* HA 1.5	*niaD1 pyrG1 areA^−^* [*barA^+^*][*pakB^ΔCRIB^* HA]	4
*pakB^ΔGBB^* HA 1.1	*niaD1 pyrG1 areA^−^* [*barA^+^*][*pakB^ΔGBB^* HA]	5
*pakB^ΔGBB^* HA 1.2	*niaD1 pyrG1 areA^−^* [*barA^+^*][*pakB^ΔGBB^* HA]	9

a Copy number was not determined for selectable markers ([*barA^+^*], [*areA^+^*] or [*pyrG1^+^*] plasmids).

The Δ*pakB pakB^+^*, Δ*pakB pakB^H204G^*, Δ*pakB pakB* ^Δ*CRIB*^ and Δ*pakB pakB* ^Δ*GBB*^ strains were generated by cotransformation of the Δ*pakB pyrG* strain with plasmids containing the appropriate mutant allele and pMT1612 (*barA^+^*). The Δ*pakB xylP(p)pakB^+^*, Δ*pakB xylP(p)pakB^H204G^*, Δ*pakB xylP(p)pakB^ΔCRIB^* and Δ*pakB xylP(p)pakB^ΔGBB^* strains were generated by transformation of Δ*pakB pyrG^−^* with the appropriate mutant allele and directly selecting for *pyrG^+^*. HA tagged strains were generated by cotransformation of G487 (*niaD^−^ pyrG^−^ areA^−^*) with the appropriate allele and pMT1612 (*barA^+^*). Southern blot analysis was used to confirm cotransformation and to determine the plasmid copy number.

The Δ*brlA* Δ*pakB* double mutant was generated by transformation of strain G526 (Δ*pkuA niaD^−^ pyrG^−^ areA^−^*) (K. Boyce and A. Andrianopoulos, unpublished) with linearized pAB5229 (A. Borneman and A. Andrianopoulos, unpublished) and selecting for pyrG^+^. Deletion of *brlA* was confirmed by Southern blot analysis. A Δ*brlA pyrG^−^* strain was isolated by plating the Δ*brlA* strain on medium containing 1 mg mL^−1^ 5-FOA supplemented with 10 mM GABA and 5 mM uracil. The Δ*brlA pyrG^−^* strain was transformed with linearized pKB6019 and Δ*brlA* Δ*pakB* double mutants selected for pyrG^+^. Deletion of *pakB* was confirmed by Southern blot analysis.

At 25°C strains were grown on *A. nidulans* minimal medium (ANM) or on synthetic dextrose (SD) medium supplemented with 10 mM ammonium sulphate ((NH_4_)_2_SO_4_) as a sole nitrogen source [36],[37]. At 37°C strains were grown on Brain Heart Infusion (BHI) medium (3.7% brain heart infusion) or Sabouraud (Sab) medium (1% mycological peptone, 2% D-glucose), malt extract (ME) medium (0.5% mycological peptone and 3% malt extract) or SD medium supplemented with 10 mM (NH_4_)_2_SO_4_. The *xylP(p)* strains were grown on carbon-free ANM plus 10 mM (NH_4_)_2_SO_4_ supplemented with either 1% glucose or 1% xylose at 25°C and on BHI ±1% xylose at 37°C.

### *In vivo* macrophage assay

J774 murine macrophages (1×10^5^) were seeded into each well of a 6 well microtitre tray containing one sterile coverslip and 2 mL of complete Dulbecco's Modified Eagle Medium (complete DMEM: DMEM, 10% fetal bovine serum, 8 mM L-glutamine and penicillin-streptomycin). Macrophages were incubated at 37°C for 24 hours before activation with 0.1 µg mL^−1^ lipopolysaccharide (LPS) from *E. coli* (Sigma). Macrophages were incubated a further 24 hours at 37°C, washed 3x in phosphate buffered saline (PBS) and 2 mL of complete DMEM medium containing 1×10^6^ conidia or yeast cells (grown at 37°C for 8 days in liquid BHI medium) was added. A control lacking conidia or yeast cells was also performed. Macrophages were incubated for 2 hours at 37°C (to allow conidia or yeast cells to be engulfed), washed once in PBS (to remove free conidia) and incubated a further 24 hours at 37°C. Macrophages were fixed in 4% paraformaldehyde and stained with 1 mg mL^−1^ fluorescent brightener 28 (calcofluor - CAL) to observe fungal cell walls. The numbers of germinated conidia was measured microscopically by counting the numbers of germinated conidia (conidia with a visible germ tube) or yeast cells in a population of approximately 100 fungal cells in macrophages. The numbers of cells with septa was measured microscopically by counting in a population of approximately 100 cells. Three independent experiments were performed. Mean and standard error of the mean values were calculated using GraphPad Prism3. To analyze the significance of the septation results, two-level nested analysis of variance (ANOVA) was performed on the data to test if the percentage of septate cells was significantly different among genotypes and also between transformants of the same genotype (http://udel.edu/~mcdonald/statnested.html) (Table S2).

Superoxide production was detected by addition of a 0.05% Nitrotetrazolium Blue Chloride (NBT) (Sigma) solution (in 0.05 M sodium phosphate buffer (pH 7.5)) to macrophages 24 hours post-infection. Macrophages were incubated for 1 hour and observed by differential interference contrast (DIC) microscopy.

To examine whether the presence of host extracts is sufficient to induce the morphological switch, LPS activated J774 murine macrophages in complete DMEM were lysed by freezing at −70°C for 20 minutes and slow thawing. The lysed extracts were added to wildtype, Δ*pakB* and Δ*pakB pakB^+^* conidia and incubated for 24 hrs at 37°C. Minus macrophage controls were also performed for comparison.

### Microscopy

*P. marneffei* strains were grown on slides covered with a thin layer of solid medium, with one end resting in liquid medium [21]. Strains were grown on ANM medium supplemented with (NH_4_)_2_SO_4_ at 25°C for 2 or 4 days. To observe conidia and yeast cells, asexually developing plates were harvested into 0.005% Tween 80 solution and filtered through Miracloth to remove hyphae. To observe conidiophores, asexually developing plates were scraped onto a coverslip containing 5 µL 0.005% Tween 80 solution. At 37°C strains were grown on BHI medium for 5 days or Sab and ME medium for 6 days. *xylP(p)* strains were grown on ANM plus 10 mM (NH_4_)_2_SO_4_ ±1% xylose at 25°C and on BHI ±1% xylose at 37°C. For germination experiments, approximately 10^6^ spores were inoculated into 300 µL of SD plus 10 mM (NH_4_)_2_SO_4_ and incubated for 15 hours at 25°C or 37°C. The rates of germination were measured microscopically by counting the numbers of germinating conidia (conidia with a visible germ tube) in a population of 100 cells. The number of conidia with 1, 2 or 3 or more germ tubes was counted in a population of 100 cells. Three independent experiments were performed. Mean and standard error of the mean values were calculated using GraphPad Prism3. Immunofluorescence microscopy for examination of the actin cytoskeleton was performed with a mouse C4 monoclonal anti-actin primary (Chemicon International, Inc.) and an ALEXA 488 rabbit anti-mouse secondary antibody (Molecular Probes). PakB HA immunofluorescence localization was performed with 3F10 rat monoclonal anti-HA primary (Boehringer Mannheim) and an ALEXA 488 goat anti-rat secondary antibody (Molecular Probes) using standard protocols [38]. Immunofluorescence microscopy controls using only primary or secondary antibodies were performed to confirm the specificity of the antibodies.

Slides were examined using differential interference contrast (DIC) and epifluorescence optics for antibody fluorescence, cell wall staining with fluorescent brightener 28 (calcofluor - CAL) or nuclear staining with Hoechst 33258 and viewed on a Reichart Jung Polyvar II microscope. Images were captured using a SPOT CCD camera (Diagnostic Instruments Inc) and processed in Adobe Photoshop^TM^.

For scanning electron microscopy (SEM), strains were grown on ANM or BHI for 10 days at 25°C or BHI for 5 days at 37°C. *xylP(p)* strains were grown on carbon-free ANM plus 10 mM (NH_4_)_2_SO_4_ supplemented with either 1% glucose or 1% xylose at 25°C for 14 days. Agar cubes containing the fungal biomass were fixed with 2.5% glutaraldehyde in PBS buffer for 2 hours, washed 3 x in PBS and postfixed with 1% osmium tetroxide for 2 hours. Samples were then washed 3 x in PBS and ethanol dehydrated by washing in increasing concentrations of ethanol. Samples were dried in a Balzers CPD 030 Critical Point Dryer and gold coated in an Edwards S150B Gold Sputter Coater. Samples were examined with a Philips XL30 FEG Field Emission Scanning Electron Microscope.

## Supporting Information

Figure S1 PakB is the *P. marneffei* Cla4 homologue. Unrooted phylogenic tree of Ste20p homologues from *Saccharomyces cerevisiae* (ScSte20), *Ashbya gossypii* (AgSte20), *Candida albicans* (CaCst20), *Ustilago maydis* (UmSmu1) and *Penicillium marneffei* (PmPakA) and Cla4p homologues from *S. cerevisiae* (ScCla4), *A. gossypii* (AgCla4), *C. albicans* (CaCla4), *U. maydis* (UmCla4) and *Clavaceps purpurea* (CpCla4). The third PAK in *S. cerevisiae*, ScSkm1, is also included. *P. marneffei* PakB (PmPakB) shows more sequence homology to Cla4p homologues than to Ste20p homologues.
(0.56 MB PDF)

Figure S2 Infection of macrophages with Δ*pakB* yeast cells results in morphological defects *in vivo*. LPS activated J774 murine macrophages infected with yeast suspensions of the wildtype (*pakB* ^+^), Δ*pakB* and Δ*pakB pakB* ^+^ strains. After 24 hours, numerous yeast cells dividing by fission were observed in macrophages infected with wildtype (*pakB* ^+^) or the Δ*pakB pakB* ^+^ strains. Macrophages infected with Δ*pakB* yeast cells contained highly branched, septate, hyphal cells. Images were captured using differential interference contrast (DIC) or with epifluorescence to observe calcofluor stained fungal cell walls (CAL). Scale bars, 20 µm.
(4.99 MB PDF)

Figure S3 *cflA* mutants produce yeast cells with wildtype morphology *in vivo*. LPS activated J774 murine macrophages infected with conidial suspensions of *cflA* ^+^, *cflA^G14V^* and *cflA^D120A^* strains. After 24 hours, numerous yeast cells dividing by fission were observed in macrophages infected with all strains. Images were captured using differential interference contrast (DIC) or with epifluorescence to observe calcofluor stained fungal cell walls (CAL). Scale bars, 10 µm.
(2.41 MB PDF)

Table S1 Percentage of *P. marneffei* septate cells 24 hrs post infection.
(0.04 MB PDF)

Table S2 Two-level nested analysis of variance (ANOVA) analysis of the percentage of septate cells in *pakB* mutants after 24 hours post-infection of macrophages. Significance 0.01, reject null hypothesis if p<0.01 (data sets are different) or accept null hypothesis if p>0.01 (data sets are not different).
(0.06 MB PDF)
