# Supplementary figures and images for: In Vivo Yeast Cell Morphogenesis Is Regulated by a p21-Activated Kinase in the Human Pathogen Penicillium marneffei
Source: PLoS Pathog. 2009 Nov 26;5(11):e1000678. doi: 10.1371/journal.ppat.1000678 (PMC2777384; doi:10.1371/journal.ppat.1000678)

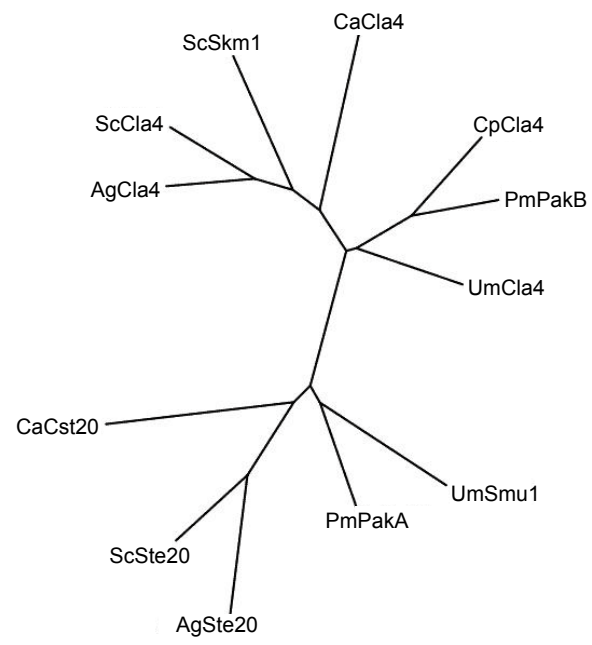

Supplement: Figure S1 — PakB is the P. marneffei Cla4 homologue. Unrooted phylogenic tree of Ste20p homologues from Saccharomyces cerevisiae (ScSte20), Ashbya gossypii (AgSte20), Candida albicans (CaCst20), Ustilago maydis (UmSmu1) and Penicillium marneffei (PmPakA) and Cla4p homologues from S. cerevisiae (ScCla4), A. gossypii (AgCla4), C. albicans (CaCla4), U. maydis (UmCla4) and Clavaceps purpurea (CpCla4). The third PAK in S. cerevisiae, ScSkm1, is also included. P. marneffei PakB (PmPakB) shows more sequence homology to Cla4p homologues than to Ste20p homologues. (0.56 MB PDF) [file ppat.1000678.s001.pdf]

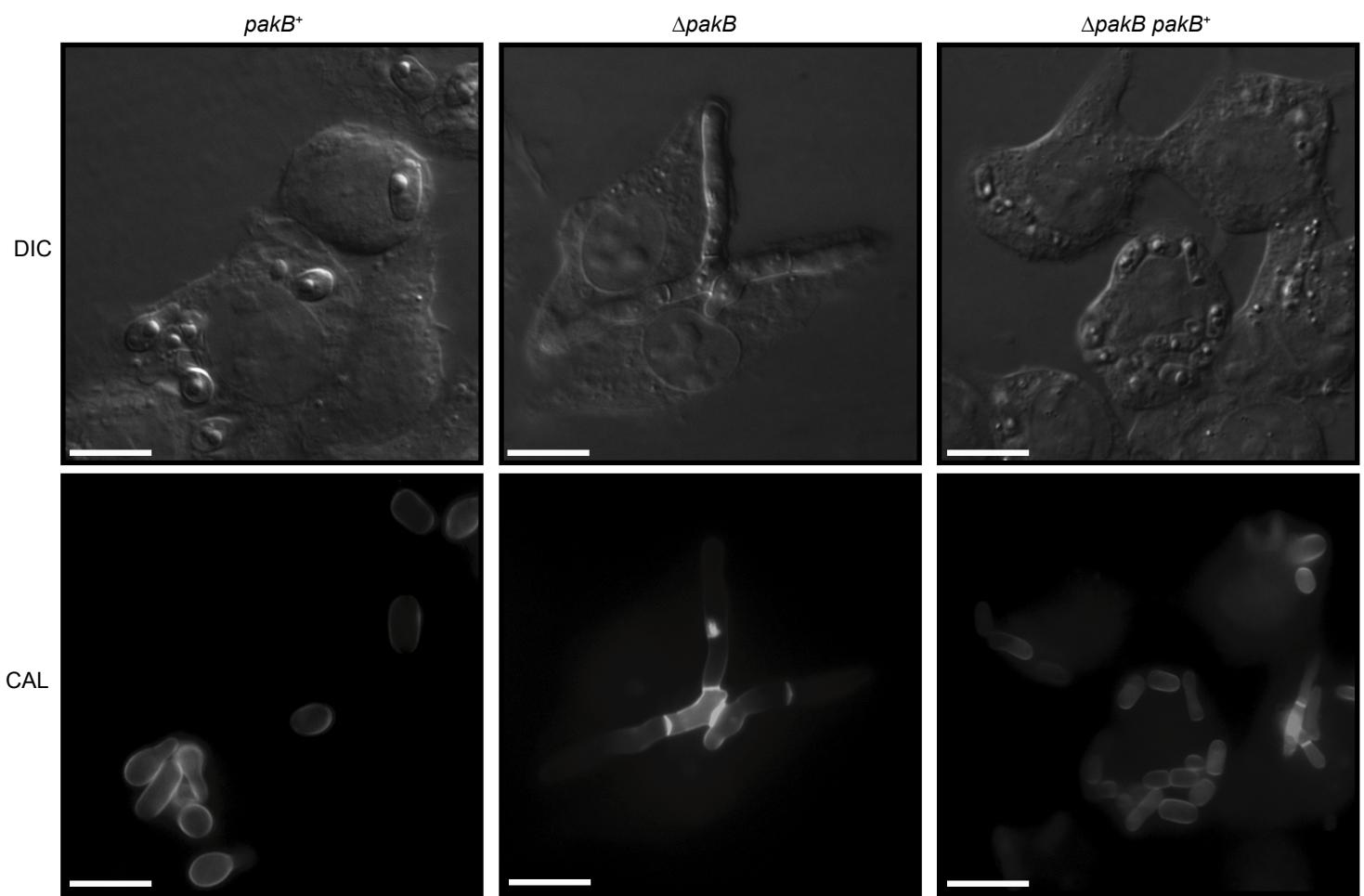

Supplement: Figure S2 — Infection of macrophages with ΔpakB yeast cells results in morphological defects in vivo. LPS activated J774 murine macrophages infected with yeast suspensions of the wildtype (pakB +), ΔpakB and ΔpakB pakB + strains. After 24 hours, numerous yeast cells dividing by fission were observed in macrophages infected with wildtype (pakB +) or the ΔpakB pakB + strains. Macrophages infected with ΔpakB yeast cells contained highly branched, septate, hyphal cells. Images were captured using differential interference contrast (DIC) or with epifluorescence to observe calcofluor stained fungal cell walls (CAL). Scale bars, 20 µm. (4.99 MB PDF) [file ppat.1000678.s002.pdf]

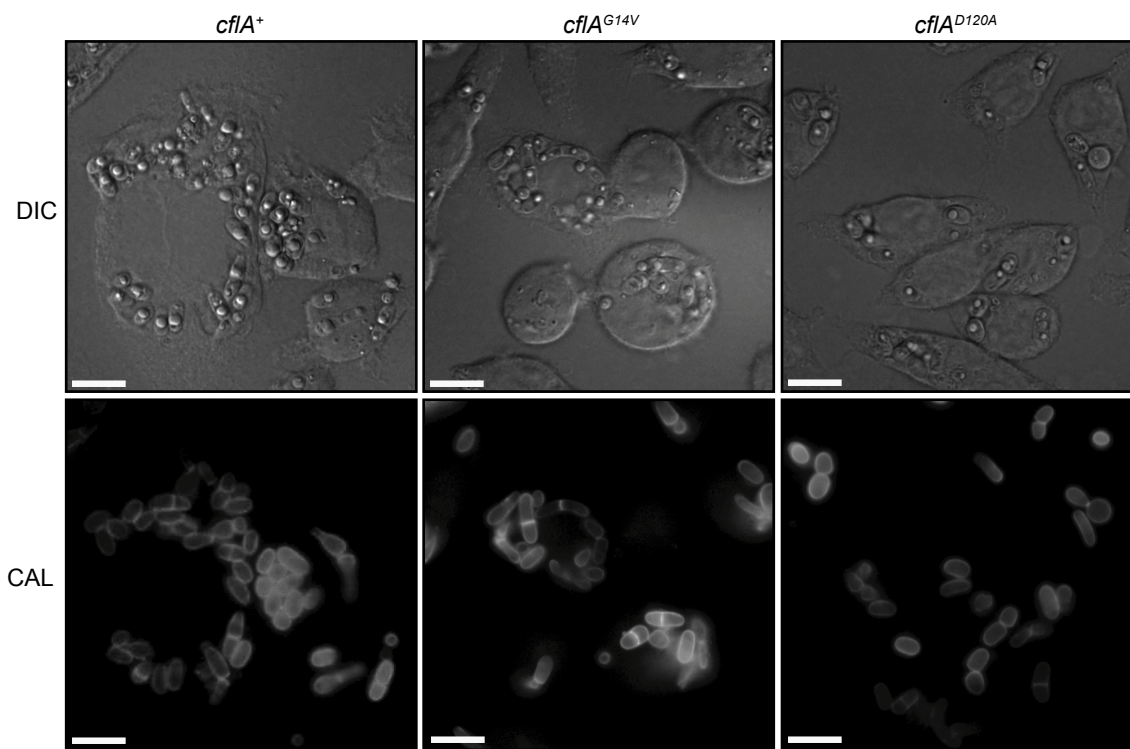

Supplement: Figure S3 — cflA mutants produce yeast cells with wildtype morphology in vivo. LPS activated J774 murine macrophages infected with conidial suspensions of cflA +, cflAG14V and cflAD120A strains. After 24 hours, numerous yeast cells dividing by fission were observed in macrophages infected with all strains. Images were captured using differential interference contrast (DIC) or with epifluorescence to observe calcofluor stained fungal cell walls (CAL). Scale bars, 10 µm. (2.41 MB PDF) [file ppat.1000678.s003.pdf]
