# Supplementary material for: In Vivo Yeast Cell Morphogenesis Is Regulated by a p21-Activated Kinase in the Human Pathogen Penicillium marneffei
Source: PLoS Pathog. 2009 Nov 26;5(11):e1000678. doi: 10.1371/journal.ppat.1000678 (PMC2777384; doi:10.1371/journal.ppat.1000678)
Supplement: Table S1 — Percentage of P. marneffei septate cells 24 hrs post infection. (0.04 MB PDF) [file ppat.1000678.s004.pdf]

**Strain Name**	**Percentage of septate cells**
pakB$^+$	17.2 ± 3.70
Δ*pakB*	71.1 ± 2.24
Δ*pakB pakB*$^+$2.1	21.6 ± 0.50
Δ*pakB pakB*$^+$2.2	16.9 ± 1.00
Δ*pakB pakB*$^+$2.3	17.5 ± 0.58
Δ*pakB pakB*$^+$2.4	22.0 ± 2.03
Δ*pakB pakB*^{H204G} 1.1	23.0 ± 2.90
Δ*pakB pakB*^{H204G} 1.3	19.1 ± 3.21
Δ*pakB pakB*^{H204G} 1.4	22.0 ± 2.09
Δ*pakB pakB*^{H204G} 2.4	24.7 ± 2.12
Δ*pakB pakB*$^{\Delta CRIB}$ 2.1	40.9 ± 4.34
Δ*pakB pakB*$^{\Delta CRIB}$ 2.3	45.4 ± 4.22
Δ*pakB pakB*$^{\Delta CRIB}$ 2.4	43.3 ± 4.94
Δ*pakB pakB*$^{\Delta CRIB}$ 2.5	40.8 ± 2.71
Δ*pakB pakB*$^{\Delta BBD}$ 2.1	60.4 ± 1.57
Δ*pakB pakB*$^{\Delta BBD}$ 2.2	60.4 ± 0.50
Δ*pakB pakB*$^{\Delta BBD}$ 2.4	66.9 ± 2.26
Δ*pakB pakB*$^{\Delta BBD}$ 2.6	61.2 ± 3.17
