# Supplementary material for: In Vivo Yeast Cell Morphogenesis Is Regulated by a p21-Activated Kinase in the Human Pathogen Penicillium marneffei
Source: PLoS Pathog. 2009 Nov 26;5(11):e1000678. doi: 10.1371/journal.ppat.1000678 (PMC2777384; doi:10.1371/journal.ppat.1000678)
Supplement: Table S2 — Two-level nested analysis of variance (ANOVA) analysis of the percentage of septate cells in pakB mutants after 24 hours post-infection of macrophages. Significance 0.01, reject null hypothesis if p<0.01 (data sets are different) or accept null hypothesis if p>0.01 (data sets are not different). (0.06 MB PDF) [file ppat.1000678.s005.pdf]

Comparison	F statistic	P value	Accept/ Reject hypothesis	Conclusion
All strains	206.97	0.000	R	Difference between genotypes
	0.63	0.810	A	No difference between transformants of the same genotype
2161<br>$\Delta pakB\ pakB^{+}$<br>$\Delta pakB\ pakB^{H204G}$	4.48	0.056	A	No difference between genotypes
	1.15	0.371	A	No difference between transformants of the same genotype
2161<br>$\Delta pakB\ pakB^{+}$<br>$\Delta pakB\ pakB^{H204G}$<br>$\Delta pakB\ pakB^{\Delta CRIB}$	91.12	0.000	R	Difference between genotypes
	0.66	0.754	A	No difference between transformants of the same genotype
2161<br>$\Delta pakB\ pakB^{+}$<br>$\Delta pakB\ pakB^{H204G}$<br>$\Delta pakB\ pakB^{\Delta GBB}$	243.65	0.000	R	Difference between genotypes
	0.80	0.629	A	No difference between transformants of the same genotype
2161<br>$\Delta pakB\ pakB^{+}$<br>$\Delta pakB\ pakB^{H204G}$<br>$\Delta pakB$	138.41	0.000	R	Difference between genotypes
	1.16	0.366	A	No difference between transformants of the same genotype
$\Delta pakB\ pakB^{\Delta CRIB}$<br>$\Delta pakB\ pakB^{\Delta GBB}$	103.90	0.000	R	Difference between genotypes
	0.42	0.856	A	No difference between transformants of the same genotype
$\Delta pakB\ pakB^{\Delta CRIB}$<br>$\Delta pakB$	132.36	0.001	R	Difference between genotypes
	0.34	0.800	A	No difference between transformants of the same genotype
$\Delta pakB\ pakB^{\Delta GBB}$<br>$\Delta pakB$	6.49	0.084	A	No difference between genotypes
	0.63	0.610	A	No difference between transformants of the same genotype
